# Essential information about nanotechnology in cardiology

**DOI:** 10.1097/MS9.0000000000002867

**Published:** 2025-01-31

**Authors:** Chukwuka Elendu, Dependable C. Amaechi, Tochi C. Elendu, Emmanuel C. Amaechi, Ijeoma D. Elendu, Janet C. Omeludike, Eunice K. Omeludike, Nwamaka C. Onubogu, Emmanuel C. Ogelle, Oluwatobi O.M. Meduoye, Praise O. Oloyede, Chiamaka P. Ezeh, Ikpembhosa J. Esangbedo, Augustina C. Adigwe, Nnachi M. Akuma, Silas U. Okafor

**Affiliations:** a Federal University Teaching Hospital, Owerri, Nigeria; b Igbinedion University, Okada, Nigeria; c Imo State University, Owerri, Nigeria; d Madonna University, Elele, Nigeria; e University of Chester, Chester, United Kingdom; f University of Portharcourt, Choba, Nigeria; g Abia State University, Uturu, Nigeria; h Blessed Specialist Hospital, Onitsha, Nigeria; i Glasgow Caledonian University, Glasgow, United Kingdom; j St. Nicholas Hospital, Lagos, Nigeria; k University College hospital, Ibadan, Nigeria; l Zaporizhzhia State Medical University, Zaporizhzhia, Ukraine

**Keywords:** cardiology, nanomedicine, nanoparticles, nanotechnology, therapeutics

## Abstract

Cardiology, as a medical specialty, addresses cardiovascular diseases (CVDs), a leading cause of global mortality. Nanomaterials offer transformative potential across key areas such as drug delivery, stem cell therapy, imaging, and gene delivery. Nanomaterials improve solubility, bioavailability, and targeted delivery in drug delivery, reducing systemic side effects. Examples include gas microbubbles, liposomal preparations, and paramagnetic nanoparticles, which show promise in treating atherosclerosis. Stem cell therapy benefits from nanotechnology through enhanced cell culture conditions and three-dimensional scaffolds that support cardiomyocyte growth and survival. Gold nanoparticles and poly(lactic-co-glycolic acid)-derived microparticles further improve stem cell viability. In imaging, nanomaterials enable advanced visualization techniques such as magnetic resonance imaging with direct labeling and optical tracking via dye-conjugated nanoparticles. In gene delivery, polymeric nanocarriers like polyethyleneimine, dendrimers, and graphene-based materials offer efficient, non-viral alternatives, with magnetic nanoparticles showing promise in targeted applications. Ongoing research highlights the potential of nanomaterials to revolutionize CVD management by improving therapeutic outcomes and enabling precision medicine. These advancements position nanotechnology as a cornerstone of modern cardiology.

## Introduction to nanotechnology

**Highlights**

- Nanotechnology represents new viable approaches for diagnosis and treatment of cardiovascular diseases, the leading cause of morbidity and mortality worldwide.
- Nanotechnology-assisted biosensing and molecular imaging can improve the sensitivity and specificity in diagnosing cardiovascular diseases.
- Nanomaterials enable targeted drug delivery or directly exert therapeutic action for the cardiovascular system based on their physicochemical properties and surface modification.

Nanotechnology, manipulating matter on an atomic and molecular scale, has emerged as a transformative field with diverse applications across various disciplines, including medicine, electronics, materials science, and energy^[1,2]^. At its core, nanotechnology involves the understanding, designing, and manipulating materials and systems at the nanoscale, typically ranging from 1 to 100 nm. This unique scale provides materials with novel properties and functionalities that differ from their bulk counterparts, enabling unprecedented control over physical, chemical, and biological phenomena^[1]^. The roots of nanotechnology can be traced back to the seminal lecture by physicist Richard Feynman in 1959, titled “There’s Plenty of Room at the Bottom,” wherein he envisioned the possibility of manipulating individual atoms and molecules to create new materials and devices with precise control^[2]^. However, it was not until the advent of scanning probe microscopy in the 1980s, particularly the development of the scanning tunneling microscope and atomic force microscope, that scientists gained the ability to visualize and manipulate individual atoms and molecules with atomic precision^[3]^. These groundbreaking advances laid the foundation for modern nanotechnology by providing researchers with the tools to explore and manipulate matter at the nanoscale. Nanotechnology research expanded rapidly throughout the 1980s and 1990s, driven by materials science, chemistry, and biology advancements^[4]^.

One of the key milestones during this period was the discovery of fullerenes, a new class of carbon molecules consisting of hollow spheres, tubes, and cages. In 1985, Harold Kroto, Robert Curl, and Richard Smalley serendipitously synthesized buckminsterfullerene (C60), a soccer ball-shaped molecule of 60 carbon atoms arranged in a spherical structure^[4]^. This discovery paved the way for developing nanomaterials with unique properties, such as high strength, conductivity, and chemical reactivity, opening up new avenues for research and applications in nanotechnology. The 1990s witnessed significant progress in nanofabrication techniques, enabling the precise manipulation and assembly of nanoscale structures. Techniques such as electron beam lithography, nanolithography, and molecular self-assembly allowed researchers to fabricate nanostructures with tailored properties and functionalities^[5]^. Concurrently, advancements in computational modeling and simulation provided valuable insights into the behavior of nanomaterials and nanoscale phenomena, facilitating the design and optimization of novel nanostructures and devices^[6]^. The turn of the 21st century marked the emergence of nanomedicine as a prominent application area of nanotechnology, with the potential to revolutionize disease diagnosis, treatment, and prevention. Nanoparticles, nanoscale drug delivery systems, and nanosensors offered new opportunities for targeted drug delivery, imaging, and diagnostics^[7]^. Researchers began exploring the use of nanomaterials for drug delivery, utilizing their unique properties, such as high surface area-to-volume ratio, tunable surface chemistry, and ability to encapsulate therapeutic agents, to improve drug efficacy, bioavailability, and safety profiles^[8]^. Concurrently, the development of nanoscale imaging agents enabled high-resolution imaging of biological structures and processes, providing valuable insights into disease pathology and treatment response^[9]^. In recent years, nanotechnology has advanced rapidly, fueled by interdisciplinary collaboration and technological innovation. Integrating nanotechnology with other fields, such as biotechnology, photonics, and information technology, has led to the development of multifunctional nanodevices and nanosystems with diverse applications. Examples include nanoscale biosensors for real-time monitoring of biomarkers, nanoelectronic devices for sensing and computing, and nanophotonic devices for light manipulation and sensing^[10]^.

Moreover, the emergence of 3D printing and nanomanufacturing techniques has facilitated the scalable production of nanomaterials and nanostructures with precise control over size, shape, and composition^[11]^. Nanotechnology promises to address some of the most pressing challenges in healthcare, energy, the environment, and beyond. Continued investment in research, infrastructure, and education is essential to unlock the full potential of nanotechnology and translate scientific discoveries into practical applications that benefit society^[12]^. By harnessing the power of the nanoscale, researchers and innovators can pave the way for a future defined by advanced technologies, improved human health, and sustainable development.

## Importance of nanotechnology in medicine

**Figure 1.**
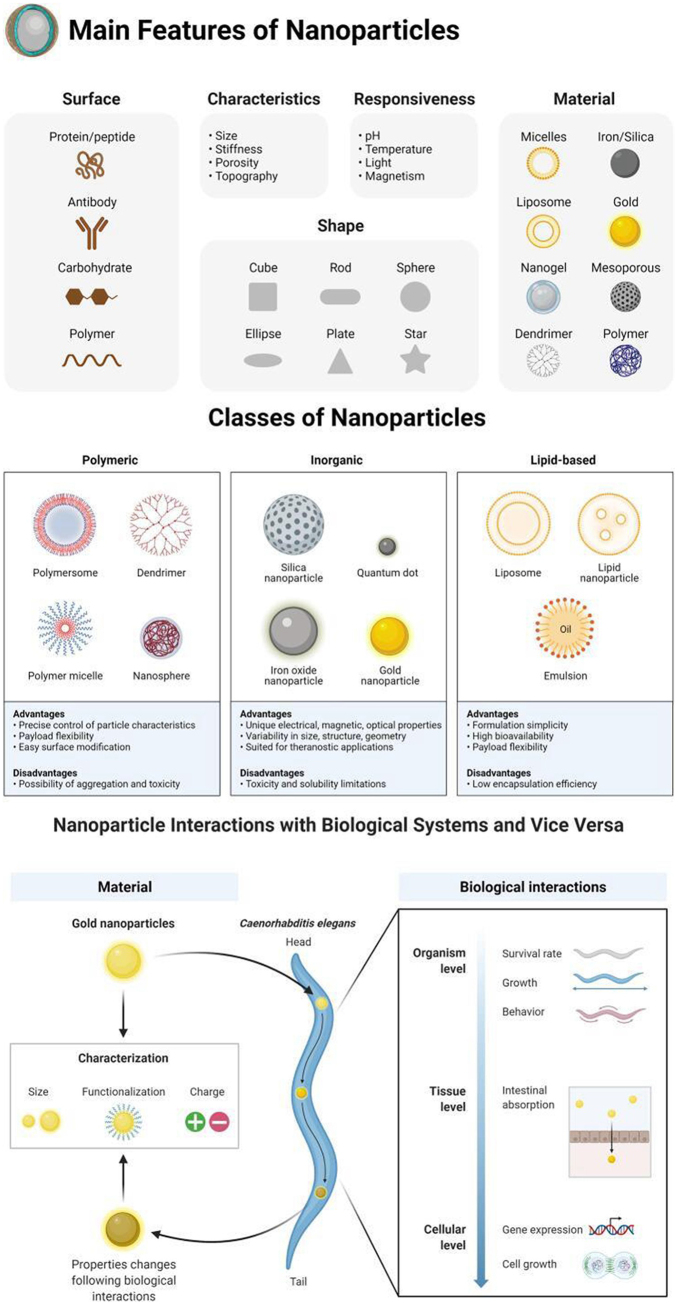
Features of nanoparticles.

**Figure 2.**
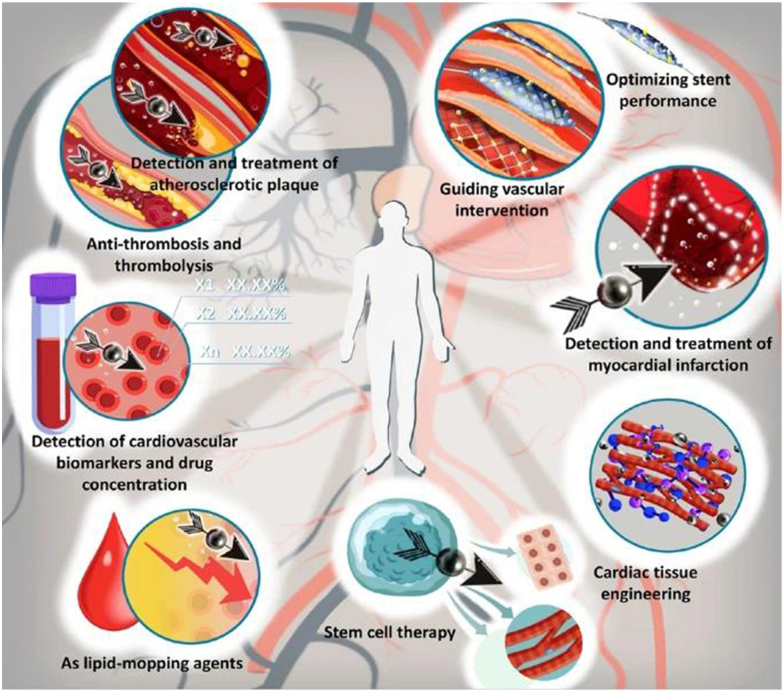
Applications of nanotechnology in cardiology.

Nanotechnology in medicine encompasses a broad spectrum of applications, each with unique advantages and challenges. One of the most significant contributions of nanotechnology to medicine is in drug delivery. Nanoparticles, with their small size and large surface area, offer a versatile platform for encapsulating and delivering therapeutic agents to target sites within the body^[13]^. This targeted drug delivery approach allows for precise localization of drugs, reducing systemic side effects and improving therapeutic efficacy^[2,14]^. Additionally, nanoparticles can be engineered to exhibit specific properties, such as sustained release kinetics or stimuli-responsive behavior, further enhancing their utility in drug delivery^[3 15]^. Nanotechnology-enabled drug delivery systems in cardiology hold immense promise for treating various cardiovascular conditions, including atherosclerosis, myocardial infarction (MI), and heart failure. For instance, nanoparticle-based formulations can be designed to deliver anti-inflammatory agents or antioxidants directly to atherosclerotic plaques, mitigating inflammation and oxidative stress within the arterial walls^[4]^. Similarly, nanoparticles loaded with cardioprotective drugs, such as statins or angiotensin-converting enzyme (ACE) inhibitors, can target ischemic myocardial tissue to limit infarct size and preserve cardiac function following a heart attack^[5]^. Table 1 displays the various applications of nanotechnology in cardiology. This table encompasses a range of innovations from nanoparticle-based drug delivery systems and advanced diagnostic imaging techniques to the development of biocompatible implants and tissue engineering solutions, highlighting the multifaceted impact of nanotechnology on cardiovascular health and treatment strategies. Figure 1 illustrates the various features and properties of nanoparticles that make them suitable for applications in cardiology. Figure 2 displays the utilization of nanotechnology in cardiovascular applications. Furthermore, nanocarriers capable of crossing the blood-brain barrier hold promise for delivering neuroprotective agents to the brain, thereby preventing secondary brain injury in patients with acute myocardial infarction (AMI) or stroke^[6]^. Beyond drug delivery, nanotechnology offers innovative solutions for cardiac imaging and diagnostics. Nanoparticles with inherent imaging properties, such as quantum dots (QDs) or gold nanoparticles, can serve as contrast agents for various imaging modalities, including magnetic resonance imaging (MRI), computed tomography (CT), and optical imaging^[7]^. These nanoparticle-based contrast agents enable high-resolution imaging of cardiovascular structures and functions, facilitating early detection and accurate diagnosis of cardiovascular diseases (CVDs)^[8]^. Moreover, nanotechnology has spurred the development of novel diagnostic platforms, such as lab-on-a-chip devices and biosensors, capable of detecting biomarkers associated with cardiovascular health and disease^[9]^. These miniaturized diagnostic tools offer rapid, point-of-care testing capabilities, allowing for timely intervention and personalized management of CVDs. Table 2 shows nanotechnology-based biosensors designed for the detection of cardiac biomarkers. These biosensors utilize various nanomaterials to achieve high sensitivity and specificity in identifying biomarkers associated with CVDs. The table highlights the types of nanomaterials used, the targeted biomarkers, detection mechanisms, and the advantages of these biosensors in clinical diagnostics. In addition to drug delivery and diagnostics, nanotechnology holds promise for regenerative medicine and tissue engineering in cardiology. Nanomaterials, such as hydrogels, nanofibers, and scaffolds, provide a supportive microenvironment for stem cell proliferation, differentiation, and integration into damaged tissues^[10]^. By mimicking the native extracellular matrix (ECM), these nanomaterial scaffolds promote tissue regeneration and repair, offering a potential solution for MI and heart failure^[11]^. Furthermore, nanotechnology-enabled approaches, such as exosome-based therapies and gene editing techniques, hold promise for enhancing the regenerative capacity of stem cells and promoting cardiac tissue repair^[12]^. Moreover, nanotechnology has opened new frontiers in the field of telemedicine and remote monitoring in cardiology. Miniaturized, wearable devices with nanosensors allow continuous monitoring of vital signs, electrocardiographic parameters, and biochemical markers, providing real-time patient cardiac health status data^[16]^. These remote monitoring systems enable early detection of arrhythmias, heart failure exacerbations, and other cardiovascular events, allowing for timely intervention and improved clinical outcomes^[13]^. Additionally, nanotechnology-enabled telemedicine platforms facilitate remote consultation, diagnosis, and management of CVDs, particularly in underserved or remote areas with limited access to healthcare services^[14]^. Despite the significant advancements and potential applications of nanotechnology in cardiology, several challenges and considerations must be addressed to realize its full clinical impact. One of the primary challenges is ensuring the safety and biocompatibility of nanomaterials used in cardiac applications. While nanomaterials offer unique properties and functionalities, their interactions with biological systems can be complex and unpredictable, raising concerns about potential toxicity, immunogenicity, and long-term effects^[15]^. Therefore, rigorous nanomaterial safety evaluation, including comprehensive toxicity studies and biocompatibility assessments, is essential to mitigate risks and ensure patient safety^[17]^. Furthermore, translating nanotechnology-based interventions from bench to bedside requires careful consideration of regulatory, ethical, and societal implications. Regulatory agencies, such as the U.S. Food and Drug Administration (FDA) and the European Medicines Agency (EMA), play a crucial role in evaluating the safety, efficacy, and quality of nanomedicine products^[18]^. However, the regulatory framework for nanotechnology-based medical devices and therapies is still evolving, posing challenges for manufacturers, researchers, and clinicians^[19]^. Moreover, ethical considerations, such as equity in access to advanced treatments and patient privacy concerns, must be addressed to ensure equitable and responsible implementation of nanotechnology in cardiology^[20]^.

The figure illustrates the modified characteristics of gold nanoparticles subsequent to ingestion by *Caenorhabditis elegans*, resulting in alterations to genetic expression and impacting various aspects of the organism’s life cycle, size, and behavior^[2]^.

This figure visually represents the diverse applications of nanotechnology in various aspects of cardiovascular medicine. It illustrates the versatility and potential impact of nanotechnology in advancing cardiac healthcare^[6]^.

**Table 1:** Applications of nanotechnology in cardiology

Type of nanotechnology	Specific applications in cardiology	Key benefits	Examples/notes
Nanoparticles	Drug delivery systems	Enhanced drug solubility, bioavailability, targeted delivery	Liposomal preparations for targeted drug delivery; polymeric nanoparticles for sustained release; gold nanoparticles for precision therapy
Nanoparticles	Imaging agents	Improved imaging contrast, specificity	Paramagnetic nanoparticles for MRI contrast; quantum dots for high-resolution imaging; dye-conjugated nanoparticles for optical imaging
Nanoparticles	Gene delivery	Efficient and safer gene transfer, non-viral methods	Polymeric nanoparticles (e.g., polyethyleneimine—PEI, dendrimers) for gene delivery; graphene-based carriers for DNA/RNA delivery; chitosan nanoparticles for enhanced stability
Nanoparticles	Stem cell therapy	Improved stem cell culture, survival, differentiation	Gold nanoparticles for enhancing stem cell survival; PLGA-derived microparticles for controlled release of growth factors; nanofiber scaffolds for 3D stem cell culture
Nanosensors	Real-time health monitoring	Continuous monitoring, early detection	Wearable devices with nanosensors for heart rate and blood pressure monitoring; implantable nanosensors for real-time glucose and lactate levels
Nanomaterials	Biocompatible implants	Enhanced integration, reduced rejection	Nanocoatings for stents to prevent restenosis; nanocomposite materials for heart valves to improve durability and compatibility
Nanotechnology-based platforms	Remote diagnosis and telemedicine	Improved access to care, real-time diagnostics	Telemedicine platforms incorporating nanosensors for remote patient monitoring; nanosensor-equipped mobile health devices for real-time ECG and blood pressure data transmission
Nanoparticles	Regenerative medicine	Promotion of tissue repair and regeneration	Bioactive nanoparticles for localized delivery of growth factors and cytokines; scaffold-based tissue engineering using nanofibers and hydrogels
Nanorobotics	Minimally invasive surgeries	Precision reduced recovery time	Nanoscale surgical instruments for precision surgeries; robotic-assisted nanoscale interventions for procedures like ablation and angioplasty
Nanotechnology	Personalized medicine	Tailored therapies, improved patient outcomes	Nanoparticle-based drug formulations tailored to genetic profiles; personalized dosing regimens based on nanosensor feedback for conditions like hypertension and arrhythmias

This table overviews nanotechnology applications in cardiology, highlighting the specific nanomaterials used, their target areas, mechanisms of action, and the resulting benefits. Applications range from drug delivery and regenerative medicine to diagnostic imaging and wearable devices, demonstrating the broad potential of nanotechnology to revolutionize cardiovascular healthcare.

**Table 2:** Nanotechnology-based biosensors for cardiac biomarkers detection

Type of biosensor	Nanomaterial used	Target biomarker(s)	Application in cardiology	Advantages	Challenges
Nanowire biosensors	Silicon nanowires	Cardiac Troponin I (cTnI)	Early detection of myocardial infarction	High sensitivity, rapid response	Fabrication complexity, integration issues
Quantum dot biosensors	Semiconductor quantum dots	B-type natriuretic peptide (BNP)	Heart failure diagnosis and monitoring	High fluorescence stability, multiplexing capability	Potential toxicity, stability under physiological conditions
Carbon nanotube biosensors	Carbon nanotubes	C-reactive protein (CRP)	Inflammation and cardiovascular risk assessment	High electrical conductivity, excellent surface area	Difficulty in functionalization, reproducibility
Gold nanoparticle biosensors	Gold nanoparticles	Myoglobin	Early myocardial injury detection	Enhanced signal amplification, biocompatibility	Cost of gold, aggregation issues
Graphene-based biosensors	Graphene	Interleukin-6 (IL-6)	Monitoring inflammatory response in heart disease	High surface area, excellent mechanical properties	Production scalability biocompatibility concerns
Magnetic nanoparticle biosensors	Iron oxide nanoparticles	N-terminal pro-b-type natriuretic peptide (NT-proBNP)	Heart failure detection and monitoring	High sensitivity, easy separation, and recovery	Magnetic interference, stability in biological systems
Polymer nanoparticle biosensors	Polymer nanoparticles	Troponin T (TnT)	Diagnosis of acute coronary syndrome	Biodegradable, flexible surface modification	Limited stability, complex synthesis
Silver nanoparticle biosensors	Silver nanoparticles	Creatine kinase-MB (CK-MB)	Acute myocardial infarction detection	Strong antimicrobial properties, enhanced conductivity	Toxicity, stability under physiological conditions
Silica nanoparticle biosensors	Silica nanoparticles	Myeloperoxidase (MPO)	Detection of plaque instability in coronary arteries	High surface area, functionalization flexibility	Biocompatibility, potential toxicity
Zinc oxide nanoparticle biosensors	Zinc oxide nanoparticles	Apolipoprotein A1 (ApoA1)	Lipid metabolism and cardiovascular disease risk assessment	High sensitivity, cost-effective	Stability under physiological conditions, toxicity
Platinum nanoparticle biosensors	Platinum nanoparticles	Fatty acid binding protein (FABP)	Early diagnosis of myocardial infarction	High catalytic activity, stability	Cost, potential toxicity
Copper nanoparticle biosensors	Copper nanoparticles	Adiponectin	Assessing metabolic syndrome and cardiovascular risk	Good conductivity, cost-effective	Oxidation issues, potential toxicity
Titanium dioxide nanoparticle biosensors	Titanium dioxide nanoparticles	Galectin-3	Fibrosis and heart failure risk assessment	High stability, biocompatibility	UV sensitivity, integration challenges
Dendritic nanoparticle biosensors	Dendrimers	ST2	Prognosis of heart failure	High loading capacity, controlled release	Complex synthesis, potential toxicity

This table overviews various nanotechnology-based biosensors used for detecting cardiac biomarkers. It includes information on the type of biosensor, the nanomaterial used, the specific biomarkers targeted, their applications in cardiology, their advantages, and the challenges they face. [Authors’ creations].

## Statement of concrete aims

- To explore various applications of nanotechnology in cardiology.
- To highlight recent advancements and prospects.

## Data collection and processing

**Literature search strategy**: A literature search was conducted to identify relevant studies related to nanotechnology applications in cardiology. Electronic databases, including PubMed, MEDLINE, Embase, Scopus, and Web of Science, were searched for articles published between 1 January 2010 and 20 April 2024. The search strategy utilized a combination of keywords and MeSH terms related to nanotechnology, cardiology, CVDs, nanoparticles, nanosensors, drug delivery systems, regenerative medicine, and telemedicine. Additionally, reference lists of relevant articles were manually reviewed to identify additional studies.

## Inclusion and exclusion criteria

Studies were included if they examined the application of nanotechnology in any aspect of cardiology, including diagnostic imaging, drug delivery, regenerative medicine, targeted therapy, remote monitoring, and telemedicine. Both experimental and clinical studies were considered, provided they directly addressed CVDs. Only articles published in peer-reviewed journals and written in English were included. Studies published between 1 January 2010 and 20 April 2024, were considered to ensure contemporary relevance and advancements in nanotechnology.

Studies were excluded if they focused solely on non-cardiac applications of nanotechnology, such as oncology or neurology. Also excluded were studies with insufficient or incomplete data regarding methodology or results, articles not published in peer-reviewed journals (e.g., conference abstracts or non-peer-reviewed reports), and non-English articles. Duplicated studies or those where the full text was unavailable were excluded, along with reviews or meta-analyses that did not provide primary data relevant to cardiology applications.

**Data extraction and synthesis**: Two reviewers independently extracted data, and any discrepancies were resolved through discussion and consensus. The following information was extracted from each included study: study design, participants/population, intervention/exposure (nanotechnology application), outcomes measured, and key findings. Data synthesis involved summarizing the main findings of the included studies and identifying common themes, trends, and gaps in the literature. The synthesized information was organized into thematic sections corresponding to the different applications of nanotechnology in cardiology.

**Quality assessment**: Due to the nature of a narrative review, formal quality assessment or risk of bias assessment of individual studies was not conducted. Instead, studies were critically appraised for relevance, methodological rigor, and contribution to the overall understanding of nanotechnology in cardiology.

## Overview of nanoparticle-based drug delivery

Nanoparticles are defined as particles with dimensions ranging from 1 to 100 nm. Due to their small size and large surface area-to-volume ratio, nanoparticles exhibit unique physical, chemical, and biological properties that can be tailored for specific medical applications. The mechanisms of nanoparticle-based drug delivery involve several key processes: encapsulation or adsorption of the drug, controlled release, targeted delivery to specific tissues or cells, and minimal off-target effects. One of the primary mechanisms by which nanoparticles enhance drug delivery is through encapsulation. Encapsulation involves incorporating therapeutic agents within a nanoparticle matrix, protecting the drug from degradation and allowing for sustained release. This method is particularly beneficial for drugs with poor solubility or stability. Polymeric nanoparticles, liposomes, and micelles are common examples of encapsulating nanoparticles. Polymeric nanoparticles, such as those made from poly(lactic-co-glycolic acid) (PLGA), provide a biodegradable and biocompatible platform for drug delivery^[1,2]^. Liposomes, composed of lipid bilayers, mimic cell membranes and are used extensively to encapsulate hydrophilic and hydrophobic drugs^[3]^. Micelles, formed by the self-assembling amphiphilic molecules, effectively solubilize poorly soluble drugs^[4]^. Another critical aspect of nanoparticle-based drug delivery is targeted delivery. Targeted delivery involves directing nanoparticles to specific cells or tissues, thereby increasing the concentration of the drug at the desired site and reducing systemic side effects. Targeting can be achieved through passive or active mechanisms. Passive targeting takes advantage of the enhanced permeability and retention effect, where nanoparticles accumulate in tumor tissues or inflamed areas due to leaky vasculature^[5]^. Active targeting, on the other hand, involves functionalizing nanoparticles with ligands such as antibodies, peptides, or small molecules that specifically bind to receptors on the target cells^[6,7]^. For instance, trastuzumab-conjugated nanoparticles have been used to target HER2-positive breast cancer cells^[8]^. Controlling nanoparticle release is another significant advantage over traditional drug delivery systems. Nanoparticles can be engineered to release their payload in a controlled manner, either through diffusion, degradation of the carrier material, or in response to external stimuli such as pH, temperature, or light^[9,10]^. This controlled release ensures a sustained therapeutic effect, reduces the frequency of administration, and improves patient compliance. For example, pH-sensitive nanoparticles release their drug payload in the acidic environment of tumor tissues, thereby enhancing the therapeutic efficacy and minimizing damage to healthy tissues^[11]^. Various types of nanoparticles are employed in drug delivery, each with unique properties and applications. Metallic nanoparticles, such as gold and silver nanoparticles, are known for their excellent stability and ease of functionalization. Gold nanoparticles, in particular, have been used for drug delivery and imaging due to their biocompatibility and tunable surface properties^[12]^. Silver nanoparticles are antimicrobial and are used in wound healing^[16]^. Polymeric nanoparticles, as mentioned earlier, offer versatility in drug encapsulation and release. PLGA nanoparticles are widely used due to their biodegradability and ability to provide sustained release^[13]^. Dendrimers, another class of polymeric nanoparticles, have a highly branched structure that allows for high drug loading capacity and precise control over drug release^[14]^. Additionally, dendrimers can be functionalized with multiple targeting moieties, enhancing their specificity for target cells^[15]^. Lipid-based nanoparticles, such as liposomes and solid lipid nanoparticles (SLNs), are popular choices for drug delivery due to their biocompatibility and ability to encapsulate a wide range of drugs. Liposomes have been successfully used to deliver anticancer drugs, such as doxorubicin and paclitaxel^[17]^. SLNs offer improved stability and controlled drug release compared to traditional liposomes^[18]^. Nanocrystals, which are pure drug particles reduced to the nanoscale, enhance the solubility and bioavailability of poorly soluble drugs. This approach is beneficial for oral drug delivery, where the dissolution rate of the drug in the gastrointestinal tract is a limiting factor^[19]^. Nanocrystals can be formulated into various dosage forms, including tablets, capsules, and suspensions, making them versatile for different routes of administration^[20]^. Carbon-based nanoparticles, such as carbon nanotubes (CNTs) and graphene, have also shown promise in drug delivery applications. CNTs can penetrate cell membranes and deliver drugs directly into the cytoplasm, while graphene-based nanoparticles offer a large surface area for drug loading and functionalization^[21,22]^. However, concerns regarding the biocompatibility and toxicity of carbon-based nanoparticles need to be addressed before widespread clinical use^[23]^. The advantages of nanoparticle-based drug delivery over traditional drug delivery systems are numerous and significant. Firstly, nanoparticles improve the solubility and stability of drugs, allowing for the formulation of therapeutics that would otherwise be challenging to deliver^[24]^. This is particularly important for hydrophobic drugs with poor bioavailability when administered using conventional methods. Secondly, nanoparticles enable targeted delivery, reducing systemic toxicity and enhancing therapeutic efficacy. Traditional drug delivery methods often result in non-specific distribution of drugs throughout the body, leading to adverse side effects and suboptimal therapeutic outcomes. By contrast, nanoparticle-based systems can be designed to deliver drugs specifically to diseased tissues or cells, minimizing off-target effects and improving patient outcomes^[25]^. Thirdly, controlled release from nanoparticles ensures a sustained therapeutic effect, reducing the need for frequent dosing and improving patient compliance. Traditional drug delivery methods often require multiple administrations to maintain therapeutic levels, which can be inconvenient and burdensome for patients. Nanoparticles can release their drug payload over an extended period, providing a more consistent therapeutic effect with fewer administrations^[26]^. Furthermore, nanoparticles offer the potential for combination therapy, where multiple drugs can be co-delivered using a single nanoparticle platform. This approach can enhance the therapeutic efficacy by targeting multiple pathways or mechanisms involved in a disease^[27]^. For example, nanoparticles co-loaded with chemotherapeutic agents and siRNA have been used to simultaneously inhibit tumor growth and suppress drug resistance^[28]^. Despite these advantages, there are challenges associated with nanoparticle-based drug delivery that need to be addressed. The biocompatibility and safety of nanoparticles are critical concerns, as the long-term effects of nanoparticle exposure on human health are not yet fully understood^[29]^. Regulatory agencies require extensive preclinical and clinical testing to ensure the safety and efficacy of nanoparticle-based therapeutics^[30]^. Moreover, the manufacturing and scalability of nanoparticles pose significant challenges. Producing nanoparticles with consistent size, shape, and functionalization at a large scale requires sophisticated techniques and stringent quality control measures^[31]^. Manufacturing nanoparticles can also be expensive, limiting their accessibility and affordability^[32]^.

## Specific applications of drug delivery systems in cardiology

Liposomal preparations are one of the most extensively studied nanoparticle-based drug delivery systems for targeted drug delivery in cardiology. Liposomes are spherical vesicles composed of lipid bilayers encapsulating hydrophilic and hydrophobic drugs. Their biocompatibility, ability to mimic biological membranes, and versatility in drug encapsulation make them an ideal platform for delivering cardiovascular therapeutics^[31]^. One of the key advantages of liposomal preparations is their capacity for targeted drug delivery. By functionalizing the surface of liposomes with targeting ligands such as antibodies, peptides, or small molecules, they can selectively bind to specific cell surface receptors that are overexpressed in diseased cardiac tissues^[32]^. This targeted approach ensures that the drug is delivered directly to the site of pathology, thereby enhancing therapeutic efficacy while minimizing systemic toxicity. For instance, liposomal formulations of doxorubicin, a chemotherapeutic agent, have been developed to target cardiomyocytes in treating cardiac sarcoma. By conjugating the liposomes with ligands specific to cardiomyocyte receptors, the drug can be selectively delivered to the heart, reducing off-target effects and improving therapeutic outcomes^[33]^. Additionally, liposomal preparations have been explored for the delivery of anti-inflammatory drugs to treat atherosclerosis. Targeted delivery of these drugs to atherosclerotic plaques can significantly reduce inflammation and plaque progression, thereby preventing adverse cardiovascular events such as MI and stroke^[34]^. Table 3 details innovative nanoplatforms designed to deliver various drugs to specific sites within the cardiovascular system. It includes information on the type of nanoparticle used, the therapeutic agents delivered, the targeted site, the mechanism of action, and the clinical benefits observed. These nanoplatforms represent significant advancements in targeted therapy, enhancing drug efficacy while minimizing side effects and improving overall patient outcomes in cardiology. Polymeric nanoparticles represent another promising class of nanoparticle-based drug delivery systems, particularly for sustained-release applications in cardiology. These nanoparticles are made from biodegradable and biocompatible polymers such as PLGA, polylactic acid (PLA), and polycaprolactone (PCL)^[35]^. The ability to control the degradation rate of these polymers allows for precise control over the release kinetics of the encapsulated drug. This sustained release mechanism ensures a prolonged therapeutic effect, reduces the frequency of drug administration, and enhances patient compliance^[36]^. In cardiology, polymeric nanoparticles have been utilized to deliver a wide range of therapeutic agents, including anti-hypertensive drugs, anticoagulants, and statins. For example, PLGA nanoparticles encapsulating amlodipine, an anti-hypertensive drug, have been developed to provide a sustained release profile, maintaining therapeutic drug levels over an extended period and improving blood pressure control in hypertensive patients^[37]^. Similarly, polymeric nanoparticles loaded with anticoagulants such as heparin have been designed to provide controlled release, reducing the risk of bleeding complications associated with traditional anticoagulant therapy^[38]^. Additionally, the sustained release of statins from polymeric nanoparticles has shown promise in managing hyperlipidemia and preventing atherosclerotic plaque formation^[39]^. Gold nanoparticles (AuNPs) have garnered significant attention in precision therapy for cardiology due to their unique optical, electronic, and chemical properties. Gold nanoparticles can be easily functionalized with various biomolecules, including drugs, peptides, and nucleic acids, allowing for highly specific and targeted delivery^[10]^. Their small size enables them to penetrate tissues and cells effectively, while their surface plasmon resonance properties facilitate imaging and diagnostic applications^[11]^. One of the most exciting applications of gold nanoparticles in cardiology is gene therapy delivery for CVDs. Gold nanoparticles can be conjugated with small interfering RNA (siRNA) or plasmid DNA to modulate gene expression in cardiomyocytes and vascular endothelial cells^[12]^. For instance, gold nanoparticles carrying siRNA targeting pro-inflammatory genes have been used to reduce inflammation in atherosclerotic plaques, thereby stabilizing the plaques and preventing rupture^[16]^. Additionally, gold nanoparticles conjugated with therapeutic genes have shown potential in regenerating damaged cardiac tissue following MI by promoting the expression of regenerative factors and inhibiting apoptotic pathways^[13]^. Moreover, gold nanoparticles have been utilized to deliver chemotherapeutic agents in treating cardiac tumors. Their ability to selectively accumulate in tumor tissues, combined with their photothermal properties, allows for the precise ablation of tumor cells with minimal damage to surrounding healthy tissue^[14]^. By irradiating the gold nanoparticles with near-infrared light, localized heating is generated, destroying cancer cells while sparing normal cardiac cells^[15]^. Another innovative application of gold nanoparticles in cardiology is their use in diagnostic imaging and theranostics. Gold nanoparticles can be conjugated with imaging agents such as fluorescent dyes or radionuclides, enhancing the visualization of cardiovascular structures and pathological changes^[17]^. This dual functionality enables the simultaneous diagnosis and treatment of CVDs, providing a comprehensive approach to patient management. For example, gold nanoparticles labeled with radiotracers have been used for positron emission tomography (PET) imaging of atherosclerotic plaques, allowing for early detection of plaque formation and monitoring therapeutic interventions^[18]^. Despite the numerous advantages and promising applications of nanoparticle-based drug delivery systems in cardiology, several challenges must be addressed to facilitate clinical translation. One of the primary challenges is ensuring the biocompatibility and safety of nanoparticles. The long-term effects of nanoparticle exposure on human health are not fully understood, and potential toxicity, immunogenicity, and unintended interactions with biological systems must be thoroughly investigated^[19]^. Rigorous preclinical and clinical testing is essential to establish the safety and efficacy of these novel drug delivery systems^[20]^. Additionally, the scalability and reproducibility of nanoparticle manufacturing pose significant challenges. Producing nanoparticles with consistent size, shape, and functionalization on a large scale requires advanced techniques and stringent quality control measures^[21]^. The high cost of manufacturing and the complexity of the production process may also limit the widespread adoption of nanoparticle-based therapeutics^[22]^. Collaboration between academia, industry, and regulatory agencies is crucial to developing standardized protocols and streamlining the manufacturing process, ensuring the availability and affordability of these advanced drug delivery systems^[23^] [^40]^.

**Table 3:** Novel nanoplatforms for drug delivery in cardiology

Nanoplatform	Drug(s) delivered	Target site	Mechanism of targeting	Advantages	Challenges
Liposomes	Doxorubicin, Paclitaxel	Atherosclerotic plaques	Passive targeting via enhanced permeability	Biocompatibility is the ability to encapsulate both hydrophobic and hydrophilic drugs	Stability issues, potential for rapid clearance
Polymeric nanoparticles	Sirolimus, Paclitaxel	Coronary artery walls	Ligand-receptor mediated targeting	Controlled release, biocompatibility	Complex synthesis, potential toxicity
Gold nanoparticles	Anti-inflammatory drugs, statins	Inflamed arterial walls	Functionalized with targeting ligands	Enhanced permeability, strong signal amplification	High cost, aggregation issues
Silica nanoparticles	Anti-cancer drugs, anti-inflammatory drugs	Tumor cells, inflamed cardiac tissues	Surface modification for active targeting	High surface area, easy functionalization	Biocompatibility, potential for toxicity
Magnetic nanoparticles	Doxorubicin, antibiotics	Ischemic heart tissues	Magnetic field-guided targeting	Noninvasive guidance, high targeting accuracy	Magnetic interference, potential toxicity
Carbon nanotubes	Anti-cancer drugs, anti-inflammatory drugs	Cardiac tumors, inflamed heart tissues	Passive targeting, functionalization	High drug loading capacity, excellent conductivity	Biocompatibility, potential toxicity
Quantum dots	Anti-inflammatory drugs	Inflamed cardiac tissues	Functionalized for specific targeting	High fluorescence stability, the potential for multiplexing	Potential toxicity, stability under physiological conditions
Dendrimers	Anti-cancer drugs, anti-inflammatory drugs	Tumor cells, inflamed cardiac tissues	Multivalency for targeted delivery	High drug loading capacity, controlled release	Complex synthesis, potential toxicity
Nanogels	Insulin, anti-cancer drugs	Cardiac tissues	Temperature/pH-responsive targeting	Biocompatibility, high drug loading capacity	Stability under physiological conditions, potential for rapid clearance
Exosomes	miRNAs, small molecules	Cardiac tissues, Ischemic heart tissues	Natural targeting ability of exosomes	Biocompatibility, natural targeting properties	Isolation and purification challenges, scalability
Polymer-lipid hybrid nanoparticles	Paclitaxel, Sirolimus	Coronary artery walls	Enhanced permeability and retention effect	Improved stability, controlled drug release	Complex synthesis and biocompatibility concerns
Ceramic nanoparticles	Anti-inflammatory drugs, chemotherapeutic agents	Tumor cells, inflamed cardiac tissues	Passive and active targeting	High stability, controlled release	Biocompatibility, potential for rapid clearance
Nanocapsules	Anti-cancer drugs, anti-inflammatory drugs	Cardiac tissues	Passive targeting, functionalization	High drug loading capacity, protection of drug payload	Complex synthesis, the potential for rapid clearance

This overviews various novel nanoplatforms used for drug delivery in cardiology. It includes information on the type of nanoplatform, the drugs delivered, the target sites, targeting mechanisms, advantages, and challenges. This table makes it easier for readers to understand nanotechnology’s diverse applications and implications in targeted drug delivery for cardiovascular diseases. It highlights the benefits and potential hurdles in implementing these advanced nanoplatforms in clinical practice. [Authors’ creations].

## Examples of successful drug delivery systems application

One of the earliest and most successful applications of nanoparticle-based drug delivery in cardiology is using liposomal doxorubicin (Doxil®) to treat cardiac sarcoma. Traditional doxorubicin administration is limited by its cardiotoxicity, which can lead to severe heart damage^[41]^. The encapsulation of doxorubicin in liposomes reduces its cardiotoxicity while maintaining its antitumor efficacy. In clinical practice, Doxil® has shown a significant reduction in cardiac side effects compared to free doxorubicin, enabling higher drug doses to be administered safely^[42]^. This case study highlights the ability of liposomal drug delivery to enhance the therapeutic index of existing drugs by minimizing their systemic toxicity. Amlodipine, a calcium channel blocker used for hypertension, typically requires daily administration due to its short half-life. PLGA nanoparticles encapsulating amlodipine have been developed to provide a sustained release profile, extending the drug’s half-life and reducing the frequency of administration. Preclinical studies have demonstrated that these nanoparticles maintain therapeutic drug levels over time, improving blood pressure control and patient compliance^[43]^. This application exemplifies how polymeric nanoparticles can be used to modify the pharmacokinetics of drugs, enhancing their clinical utility. Gold nanoparticles have been explored for their photothermal properties in treating CVDs such as atherosclerosis. This approach conjures gold nanoparticles with targeting ligands that bind to atherosclerotic plaques. The gold nanoparticles generate localized heat upon exposure to near-infrared light, ablating the plaque and restoring normal blood flow. Preclinical studies have shown that this method effectively reduces plaque size and inflammation without damaging surrounding healthy tissue^[44]^. This case study demonstrates the potential of gold nanoparticles in providing a minimally invasive, targeted treatment for CVDs.

## Drug delivery systems trials and outcomes

CER-001 is a liposomal formulation designed to deliver siRNA specifically to atherosclerotic plaques. The siRNA targets and downregulates pro-inflammatory genes involved in plaque formation and progression. A Phase I clinical trial has demonstrated the safety and feasibility of this approach, showing promising results in reducing plaque size and inflammation markers^[45]^. Ongoing Phase II trials are assessing the efficacy of CER-001 in larger patient populations, with the potential to offer a novel therapeutic strategy for atherosclerosis that directly targets the underlying molecular mechanisms of the disease. Polymeric nanoparticles are being investigated for their ability to deliver therapeutic agents to the heart following MI. One example is using PLGA nanoparticles encapsulating anti-apoptotic and regenerative peptides to promote cardiac repair and reduce scar formation. Early-phase clinical trials currently evaluate these nanoparticles’ safety and preliminary efficacy in patients with an MI^[46]^. If successful, this approach could significantly improve recovery and outcomes for MI patients by enhancing tissue regeneration and reducing the incidence of heart failure. A novel clinical trial explores using gold nanoparticles conjugated with gene therapy vectors to treat heart failure. This trial aims to deliver genes encoding for proteins that enhance cardiac contractility and reduce fibrosis directly to the myocardium. Preclinical studies have shown that this approach can improve cardiac function and reduce pathological remodeling in animal models of heart failure^[47]^. The ongoing clinical trial is assessing this strategy’s safety and therapeutic potential in human patients, hoping to offer a new treatment modality for heart failure that addresses the disease at the genetic level. Liposomal formulations of cyclosporine, an immunosuppressive drug used in heart transplant patients, are being evaluated to reduce nephrotoxicity and improve graft survival. Cyclosporine is known for its potential to cause kidney damage. It can be mitigated by encapsulating the drug in liposomes to enhance its targeted delivery to the heart and reduce off-target effects. Initial clinical trials have demonstrated improved pharmacokinetics and reduced renal toxicity in transplant recipients, leading to ongoing Phase II trials further to evaluate the long-term benefits and safety of liposomal cyclosporine^[48]^. A clinical trial is investigating the use of polymeric nanoparticles to deliver anti-inflammatory and pro-angiogenic drugs for treating peripheral artery disease (PAD). These nanoparticles aim to enhance blood flow and tissue regeneration in ischemic limbs, potentially reducing the need for invasive procedures such as angioplasty or bypass surgery. Early-phase trials have shown improved limb perfusion and reduced inflammation in patients with PAD, and ongoing trials are focused on confirming these benefits in larger cohorts^[49]^.

## Diagnostic imaging

Nanoparticle-based contrast agents for MRI and CT have emerged as powerful tools for enhancing the quality and specificity of medical images. Traditional contrast agents, such as gadolinium-based compounds for MRI and iodine-based compounds for CT, have limitations, including toxicity, rapid clearance from the body, and suboptimal contrast enhancement^[50]^. Nanoparticles, with their unique physicochemical properties, offer solutions to these challenges by providing higher contrast, longer circulation times, and reduced toxicity. Superparamagnetic iron oxide nanoparticles (SPIONs) have been extensively studied and utilized for MRI. These nanoparticles generate strong magnetic fields that enhance the relaxation rates of nearby water protons, thereby increasing the contrast of the MRI images. SPIONs can be functionalized with various surface coatings to improve their biocompatibility and target specific tissues or cellular markers. For example, dextran-coated SPIONs have been used to visualize macrophage activity in atherosclerotic plaques, providing valuable insights into plaque composition and stability^[51]^. Moreover, SPIONs have been employed to monitor stem cell therapy in cardiac regeneration, allowing researchers to track the migration and integration of transplanted cells in real-time^[52]^. Gold nanoparticles (AuNPs) have also been explored as contrast agents for CT imaging. Due to their high atomic number and electron density, AuNPs provide superior contrast enhancement compared to traditional iodine-based agents. Additionally, gold nanoparticles can be engineered to have controlled sizes and shapes, optimizing their biodistribution and pharmacokinetics for targeted imaging. Functionalization of AuNPs with targeting ligands, such as antibodies or peptides, enables precise localization to specific tissues or pathological sites, enhancing the specificity of the imaging^[53]^. For instance, AuNPs conjugated with a peptide that targets integrin receptors have been used to selectively image angiogenesis in MI models, aiding in the assessment of neovascularization and tissue repair^[54]^. Table 4 outlines the use of various nanomaterials in noninvasive molecular imaging techniques to diagnose CVDs. It includes details on specific nanomaterials, their imaging modalities, targeted cardiovascular conditions, mechanisms of action, and their advantages in improving diagnostic accuracy and patient outcomes. QDs represent another significant advancement in optical imaging, offering unique advantages such as high brightness, photostability, and tunable emission wavelengths. These semiconductor nanocrystals can be excited by a wide range of light wavelengths, emitting bright and stable fluorescence that can be detected with high sensitivity. QDs are particularly useful for multiplexed imaging, where multiple biomarkers can be simultaneously visualized using QDs with different emission spectra. This capability is invaluable in cardiology, where understanding the complex interplay of various cellular and molecular processes is crucial. For example, QDs have been employed to label and track endothelial cells in vascular biology and angiogenesis studies. By conjugating QDs with antibodies against endothelial cell markers, researchers can visualize the dynamics of endothelial cell behavior and their interactions with other cell types during the formation of new blood vessels^[55]^. Additionally, QDs have been used to study the trafficking of inflammatory cells, such as monocytes and macrophages, in CVDs, providing insights into the role of inflammation in disease progression^[56]^. Despite their promise, the clinical translation of nanoparticle-based contrast agents and QDs faces several challenges. One major concern is the potential toxicity of these nanomaterials. For instance, while SPIONs are generally considered safe, high doses or prolonged exposure may lead to iron overload and oxidative stress, damaging tissues^[57]^. Similarly, the core materials of QDs, such as cadmium, are toxic, and the degradation of QDs in vivo can release these harmful components^[58]^. To address these issues, researchers are developing biocompatible coatings and biodegradable formulations to minimize toxicity and enhance the safety profile of these imaging agents. Another challenge is the complexity of the regulatory approval process for nanomaterials. Regulatory agencies, such as the FDA and EMA, require extensive preclinical and clinical data to evaluate the safety and efficacy of new imaging agents. The unique properties of nanoparticles necessitate the development of new testing protocols and guidelines to assess their interactions with biological systems, biodistribution, and potential long-term effects^[59]^. Collaborative efforts between researchers, industry, and regulatory bodies are essential to streamline the approval process and ensure the safe implementation of nanoparticle-based imaging technologies in clinical practice. In addition to safety and regulatory challenges, there are technical hurdles related to the synthesis and functionalization of nanoparticles. Achieving uniform particle size and shape, consistent surface functionalization and scalable production are critical for the reproducibility and reliability of nanoparticle-based imaging agents. Advances in nanofabrication techniques, such as microfluidics and self-assembly, are being explored to overcome these challenges and produce high-quality nanoparticles with precise control over their properties^[60]^. Moreover, integrating nanoparticle-based contrast agents and QDs into existing imaging modalities requires the development of specialized imaging protocols and equipment. For example, areas of active research include optimizing MRI sequences to maximize the contrast provided by SPIONs or designing optical imaging systems that can efficiently detect and discriminate the emission spectra of QDs^[11]^. The successful integration of these technologies into clinical workflows will depend on close collaboration between engineers, radiologists, and clinicians. Despite these challenges, the potential benefits of nanoparticle-based imaging techniques in cardiology are substantial. Enhanced imaging capabilities can improve the early detection and diagnosis of CVDs, allowing for timely interventions and better patient outcomes. For instance, the ability to visualize and characterize atherosclerotic plaques with high precision can aid in the risk stratification of patients and guide the selection of appropriate therapeutic strategies^[12]^. Similarly, monitoring stem cell therapy and tissue regeneration using nanoparticle-based imaging agents can provide valuable feedback on the efficacy of these treatments and inform the optimization of therapeutic protocols^[16]^. In addition to diagnostic applications, nanoparticle-based imaging techniques have potential therapeutic implications. Theranostic nanoparticles, which combine diagnostic and therapeutic functions, are being developed to deliver targeted therapy while simultaneously monitoring treatment response. For example, SPIONs loaded with chemotherapeutic agents or anti-inflammatory drugs can be directed to atherosclerotic plaques, where their therapeutic effects can be visualized and quantified using MRI^[13]^. This approach enables real-time treatment efficacy assessment and personalized adjustments to the therapeutic regimen. QDs also hold promise for theranostic applications in cardiology. By conjugating QDs with therapeutic agents and targeting moieties, researchers can achieve precise delivery of drugs to specific cells or tissues while using the fluorescent properties of QDs to monitor drug release and distribution^[14]^. This capability is particularly valuable in CVDs, where targeted therapies can minimize off-target effects and enhance therapeutic efficacy. The continuous evolution of imaging modalities has led to significant improvements in spatial and temporal resolution, enabling clinicians to visualize cardiac structures with unprecedented detail and clarity. High-resolution imaging techniques, such as cardiac MRI and computed tomography angiography (CTA), offer exquisite anatomical delineation of the heart and great vessels, facilitating accurate diagnosis and treatment planning^[61]^. For example, cardiac MRI with high-resolution sequences allows for the visualization of myocardial tissue characteristics, including MI, fibrosis, and edema, with superior contrast and spatial resolution^[62]^. Similarly, CTA provides detailed three-dimensional reconstructions of the coronary arteries, enabling the precise localization of stenotic lesions and the assessment of coronary artery disease severity^[63]^. Moreover, advancements in imaging technology have improved the specificity of diagnostic imaging modalities, allowing for the differentiation of various cardiac pathologies based on their unique imaging characteristics. Contrast-enhanced imaging techniques, such as myocardial perfusion imaging and late gadolinium enhancement MRI, enable the detection of myocardial ischemia, scar tissue, and microvascular dysfunction with high sensitivity and specificity^[64]^. Additionally, molecular imaging approaches, such as PET and single-photon emission computed tomography (SPECT), allow for the noninvasive visualization of molecular processes underlying CVDs, such as inflammation, angiogenesis, and apoptosis^[65]^. These targeted imaging techniques provide valuable insights into disease mechanisms and help guide personalized treatment strategies tailored to individual patient profiles. Early detection and diagnosis of CVDs are critical for timely interventions and improved patient outcomes. Diagnostic imaging modalities play a central role in the early detection of cardiac abnormalities, allowing for the identification of subclinical disease states and the initiation of preventive measures before the onset of symptomatic disease. Screening programs utilizing noninvasive imaging techniques, such as echocardiography and coronary artery calcium scoring, enable the early detection of structural and functional abnormalities, such as left ventricular hypertrophy, valvular dysfunction, and coronary artery calcifications, in asymptomatic individuals at risk for CVDs^[66]^. Early identification of these risk factors allows for implementing lifestyle modifications, pharmacological interventions, and risk factor management strategies to reduce the progression of CVD and prevent adverse cardiovascular events. Furthermore, advancements in imaging technology have enabled the early diagnosis of acute cardiovascular conditions, such as AMI and acute coronary syndromes, facilitating prompt triage and treatment in the emergency setting. Rapid imaging protocols, such as coronary CT angiography and cardiac MRI, provide rapid and accurate assessment of coronary artery patency, myocardial perfusion, and cardiac function, allowing clinicians to rapidly identify and triage patients presenting with acute chest pain or suspected acute coronary syndrome^[67]^. Early diagnosis and risk stratification based on imaging findings enable timely reperfusion therapies, such as percutaneous coronary intervention or thrombolytic therapy, which are crucial for salvaging ischemic myocardium and improving patient outcomes^[68]^. In addition to their diagnostic utility, imaging modalities are pivotal in guiding therapeutic interventions and monitoring treatment response in CVDs. Image-guided interventions, such as catheter-based coronary angioplasty and transcatheter aortic valve replacement, rely on real-time imaging guidance to ensure precise device placement and optimal procedural outcomes^[69]^. Furthermore, advanced imaging techniques, such as strain imaging and myocardial tagging MRI, allow for the quantitative assessment of cardiac function and myocardial mechanics, providing valuable prognostic information and guiding therapeutic decision-making in patients with heart failure, cardiomyopathies, and myocardial ischemia^[10]^. Despite the numerous advancements in diagnostic imaging technology, several challenges and limitations persist. The availability and accessibility of advanced imaging modalities may be limited in certain geographical regions or healthcare settings, posing barriers to equitable healthcare delivery and patient access to optimal diagnostic services. Moreover, the high cost of sophisticated imaging equipment and the need for specialized training and expertise in interpreting imaging studies may hinder the widespread adoption and utilization of advanced imaging techniques, particularly in resource-constrained settings^[11]^. One of the primary challenges in diagnostic imaging is the limited resolution and sensitivity of current imaging modalities, particularly for detecting early-stage cardiovascular abnormalities and subtle structural changes. Despite advancements in imaging technology, certain cardiac pathologies, such as microvascular dysfunction, early-stage atherosclerosis, and small MIs, may not be adequately visualized or accurately characterized using conventional imaging techniques^[1]^. Improving the resolution and sensitivity of diagnostic imaging modalities is essential for early detection, accurate diagnosis, and timely intervention in CVDs, ultimately leading to improved patient outcomes and reduced morbidity and mortality. Another significant limitation of current imaging techniques is their inability to provide real-time, dynamic imaging of cardiac function and physiology, particularly under physiological stress conditions. Traditional imaging modalities, such as echocardiography and cardiac MRI, offer static snapshots of cardiac structure and function at rest but may not capture dynamic changes in myocardial contractility, perfusion, and mechanical strain during exercise or pharmacological stress testing^[2]^. Real-time imaging techniques capable of dynamically assessing cardiac function and physiology are needed to understand CVD pathophysiology better and guide personalized treatment strategies tailored to individual patient responses. Moreover, current imaging techniques often lack specificity in distinguishing between benign and malignant cardiac lesions or differentiating between various cardiac pathologies with similar imaging appearances. Differential diagnosis of cardiac tumors, inflammatory conditions, and benign structural abnormalities based on imaging findings alone can be challenging. It may require invasive diagnostic procedures, such as biopsy or surgical excision, for definitive diagnosis^[3]^. Improving the specificity of diagnostic imaging modalities by developing novel contrast agents, molecular imaging probes, and advanced image analysis algorithms is essential for accurate diagnosis and risk stratification in CVDs. In addition to technical limitations, practical challenges are associated with the accessibility, affordability, and standardization of diagnostic imaging techniques, particularly in resource-limited healthcare settings. High-cost imaging equipment, specialized training requirements for imaging technologists and interpreters, and infrastructure limitations may restrict access to advanced imaging modalities in underserved communities, rural areas, and low-income countries^[4]^. Addressing these disparities in healthcare access and promoting equitable distribution of diagnostic imaging resources are essential for improving healthcare outcomes and reducing health inequities globally. To address these challenges and advance the field of diagnostic imaging in cardiology, several research and development needs must be prioritized. First and foremost, there is a need for continued investment in basic and translational research aimed at developing innovative imaging technologies and techniques that overcome the limitations of current modalities^[5]^. This includes developing novel imaging contrast agents, such as molecular probes targeting specific biomarkers of CVDs, and integrating artificial intelligence (AI) and machine learning (ML) algorithms for automated image analysis and interpretation^[6]^. Furthermore, collaborative efforts between academia, industry, and regulatory agencies are essential for accelerating the translation of research findings into clinical practice and ensuring emerging imaging technologies’ safety, efficacy, and regulatory compliance^[7]^. Multicenter clinical trials and validation studies are needed to establish novel imaging modalities’ clinical utility and diagnostic accuracy in diverse patient populations and clinical settings^[8]^. Standardized imaging protocols and reporting guidelines should also be developed to facilitate data interoperability, comparability, and reproducibility across different imaging platforms and healthcare institutions^[9]^. Education and training initiatives to enhance the imaging literacy of healthcare providers, researchers, and patients are also critical for promoting the adoption and appropriate utilization of advanced imaging techniques in clinical practice^[10]^. This includes developing educational curricula, certification programs, and continuing medical education opportunities focused on cardiovascular imaging modalities, interpretation skills, and clinical decision-making^[11]^. Moreover, patient engagement and empowerment strategies, such as shared decision-making and health literacy promotion, can help improve patient understanding of diagnostic imaging procedures, risks, and benefits, leading to more informed healthcare choices and improved treatment adherence^[12^] [^70]^.

**Table 4:** Nanomaterial-based noninvasive molecular imaging for cardiovascular diagnosis

Nanomaterial	Imaging modality	Targeted biomarkers/cells	Mechanism of targeting	Advantages	Challenges
Gold nanoparticles	CT, photoacoustic Imaging	Atherosclerotic plaques, macrophages	Functionalized with targeting ligands	High contrast, stability, biocompatibility	Cost, potential for aggregation
Iron oxide nanoparticles	MRI	Macrophages, inflammatory cells	Magnetic field-guided targeting	High-resolution, non-toxic, biocompatible	Magnetic interference, stability issues
Quantum dots	Fluorescence imaging	Endothelial cells, cardiac myocytes	Surface modification for targeting	High fluorescence, multiplexing ability	Potential toxicity, photobleaching
Silica nanoparticles	PET, MRI	Tumor cells, inflamed tissues	Surface modification for targeting	High surface area, easy functionalization	Biocompatibility, potential for toxicity
Carbon nanotubes	Raman spectroscopy, MRI	Tumor cells, inflamed cardiac tissues	Functionalized for targeting	High conductivity, strong Raman signal	Biocompatibility, potential toxicity
Liposomes	SPECT, PET	Cardiac tissues, ischemic regions	Encapsulation of imaging agents	Biocompatibility, ability to carry large payloads	Stability, potential for rapid clearance
Dendrimers	MRI, PET	Cardiac myocytes, endothelial cells	Multivalency for targeted delivery	High drug loading capacity, controlled release	Complex synthesis, potential toxicity
Polymeric nanoparticles	MRI, ultrasound Imaging	Inflamed cardiac tissues	Ligand-receptor mediated targeting	Controlled release, biocompatibility	Complex synthesis, potential toxicity
Nanogels	MRI, fluorescence Imaging	Cardiac tissues, ischemic areas	Temperature/pH-responsive targeting	Biocompatibility, high drug loading capacity	Stability under physiological conditions
Exosomes	MRI, ultrasound Imaging	Cardiac tissues, ischemic regions	Natural targeting ability of exosomes	Biocompatibility, natural targeting properties	Isolation and purification challenges
Ceramic nanoparticles	MRI, CT	Tumor cells, inflamed cardiac tissues	Surface modification for targeting	High stability, controlled release	Biocompatibility, potential for rapid clearance

This table provides an in-depth overview of nanomaterials utilized in noninvasive molecular imaging to diagnose cardiovascular conditions. It includes information on the type of nanomaterial, the imaging modality used, targeted biomarkers or cells, targeting mechanisms, advantages, and challenges. This table enhances the understanding of how nanotechnology can be applied to improve the specificity, resolution, and effectiveness of cardiovascular diagnostic imaging. It highlights the unique benefits each nanomaterial offers and the potential limitations that must be addressed to realize their clinical potential fully. [Authors’ creations].

## Nanotechnology in gene delivery

Polymeric nanoparticles have emerged as versatile and promising vehicles for gene delivery due to their tunable physicochemical properties, biocompatibility, and ability to protect nucleic acids from degradation in biological environments^[1]^. These nanoparticles are typically composed of biodegradable polymers, such as polyethyleneimine, PLGA, chitosan, and dendrimers, which can encapsulate or complex with nucleic acids, including plasmid DNA, small interfering RNA (siRNA), and microRNA (miRNA), for targeted delivery to specific cells or tissues^[2]^. The design and formulation of polymeric nanoparticles can be tailored to optimize their transfection efficiency, stability, and biocompatibility, making them attractive candidates for gene therapy applications in cardiology. One of the key advantages of polymeric nanoparticles for gene delivery is their safety profile compared to viral vectors. Viral vectors, such as adenoviruses, retroviruses, and lentiviruses, have been associated with various safety concerns, including insertional mutagenesis, immunogenicity, and inflammatory responses, which can limit their clinical utility and pose risks to patient safety^[3]^.

In contrast, polymeric nanoparticles are inherently non-immunogenic. They can be engineered to minimize off-target effects and enhance target cell specificity through surface modification with targeting ligands, such as antibodies, peptides, or aptamers^[4]^. Additionally, the biodegradable nature of polymeric nanoparticles allows for controlled and sustained release of therapeutic genes, reducing the risk of systemic toxicity and improving the safety profile of gene delivery systems^[5]^. Furthermore, polymeric nanoparticles offer several advantages over viral vectors regarding cargo capacity, flexibility, and scalability. Viral vectors have limited cargo capacity, typically accommodating gene inserts of up to 8−10 kb, which may restrict the delivery of large or multiple genes for complex therapeutic applications^[6]^.

In contrast, polymeric nanoparticles can accommodate larger payloads of nucleic acids, including plasmids encoding multiple genes or regulatory elements, enabling the delivery of more sophisticated gene constructs for modulating complex biological pathways implicated in CVDs^[7]^. Moreover, the synthesis and production of polymeric nanoparticles are relatively straightforward and scalable, allowing for cost-effective manufacturing and large-scale production to meet clinical demand^[8]^. In addition to their safety and scalability, polymeric nanoparticles offer several unique features that make them attractive for targeted gene delivery in cardiology. These nanoparticles can be engineered to overcome biological barriers, such as the ECM, cell membranes, and endosomal compartments, which can impede the intracellular delivery of nucleic acids^[9]^. Surface modification of polymeric nanoparticles with cell-penetrating peptides or pH-sensitive polymers can enhance cellular uptake and endosomal escape, facilitating the efficient delivery of therapeutic genes to target cells within the cardiovascular system^[10]^.

Moreover, the tunable properties of polymeric nanoparticles, including size, shape, surface charge, and degradation kinetics, allow for precise control over their pharmacokinetics, biodistribution, and release kinetics, optimizing their therapeutic efficacy and minimizing off-target effects^[11]^. Despite these advantages, several challenges and considerations must be addressed to advance the clinical translation of polymeric nanoparticles for gene delivery in cardiology. One of the key challenges is nanoparticle design and formulation optimization to achieve efficient gene transfection in vivo while minimizing cytotoxicity and immune responses^[12]^. Developing biocompatible and biodegradable polymers with optimal physicochemical properties and optimizing nanoparticle size, surface charge, and surface modification strategies are critical for enhancing the efficacy and safety of polymeric nanoparticle-based gene delivery systems^[16]^. Moreover, delivering therapeutic genes to specific cell types or tissues within the cardiovascular system remains a significant challenge due to cardiac tissues’ complex and heterogeneous nature and physiological barriers, such as the blood-brain and endothelial barriers^[13]^. Strategies for enhancing the targeting specificity and tissue penetration of polymeric nanoparticles, such as using tissue-specific ligands or stimuli-responsive materials, are actively being explored to overcome these challenges and improve the therapeutic outcomes of gene therapy in cardiology^[14]^.

## Stem cell therapy enhancement

One of the key challenges in stem cell therapy is the inability to monitor the fate and behavior of transplanted cells in real time following administration. Nanoparticle-based labeling and tracking strategies offer a noninvasive and sensitive approach to monitoring stem cell migration, homing, engraftment, and differentiation in vivo, providing valuable insights into their therapeutic mechanisms and optimizing treatment strategies^[1]^. Various types of nanoparticles, including superparamagnetic iron oxide nanoparticles (SPIONs), QDs, and gold nanoparticles (AuNPs), have been employed for labeling and tracking stem cells in preclinical and clinical studies^[2]^. SPIONs are widely used as MRI contrast agents for labeling and tracking stem cells due to their biocompatibility, high magnetic susceptibility, and ability to generate signal voids on MRI images^[3]^. Stem cells can internalize these nanoparticles via endocytosis and remain trapped within the cells, allowing for long-term tracking of labeled cells in vivo^[4]^. QDs, on the other hand, are semiconductor nanoparticles that exhibit unique optical properties, including size-dependent fluorescence emission and high photostability, making them ideal probes for fluorescence imaging of stem cells^[5]^. QDs can be conjugated to cell surface markers or intracellular targets to visualize and track the migration and differentiation of stem cells in real time^[6]^. Gold nanoparticles have also been explored for stem cell labeling and tracking, offering advantages such as ease of synthesis, tunable surface chemistry, and compatibility with various imaging modalities, including CT and photoacoustic imaging^[7]^. These nanoparticles can be functionalized with targeting ligands or imaging agents to enhance their specificity and sensitivity for stem cell tracking applications^[8]^. Moreover, gold nanoparticles can serve as theranostic agents, combining diagnostic imaging with therapeutic interventions, such as photothermal therapy or drug delivery, to enhance the efficacy of stem cell-based therapies^[9]^. Overall, nanoparticle-based labeling and tracking techniques enable the noninvasive monitoring of stem cells in vivo, optimizing stem cell delivery routes, dosages, and timing for maximal therapeutic benefit in cardiovascular regeneration^[10]^. However, several challenges must be addressed, including nanoparticle toxicity, immunogenicity, and clearance, to ensure nanoparticle-labeled stem cell therapies’ safety and clinical translation^[11]^. In addition to tracking stem cells, nanotechnology offers innovative solutions for enhancing the survival, retention, and therapeutic efficacy of transplanted cells by developing biomimetic scaffolds and nanoparticle-mediated delivery of growth factors^[12]^. Biomimetic scaffolds provide structural support and biochemical cues to promote cell adhesion, proliferation, and differentiation, mimicking the native ECM environment^[16]^. These scaffolds can be engineered at the nanoscale to precisely control their mechanical properties, topography, and bioactive signaling molecules, enhancing their compatibility with stem cells and facilitating tissue regeneration^[13]^. Nanofibrous scaffolds, for example, are fabricated using electrospinning techniques to generate nanoscale fibers resembling the native ECM, which can promote cell attachment, migration, and tissue integration^[14]^. These scaffolds can be functionalized with bioactive peptides, growth factors, or nanoparticles to enhance their regenerative properties and guide stem cell fate toward desired lineages^[15]^. Similarly, nanoparticle-based delivery systems offer a versatile platform for the controlled release of growth factors and bioactive molecules to create a pro-regenerative microenvironment and enhance the therapeutic potential of stem cells^[17]^. Nanoparticles can be loaded with growth factors, such as vascular endothelial growth factor (VEGF), fibroblast growth factor, or insulin-like growth factor (IGF), and embedded within scaffolds or injected directly into the target tissue to stimulate angiogenesis, vasculogenesis, and tissue repair^[18]^. These growth factor-loaded nanoparticles can provide sustained release kinetics, prolonging their bioactivity and therapeutic effects while minimizing systemic side effects and dosage-related toxicity^[19]^. Moreover, nanoparticles can be engineered to respond to specific stimuli, such as pH, temperature, or enzymatic activity, enabling the on-demand release of growth factors in response to physiological cues^[20]^. Furthermore, nanotechnology enables the development of multifunctional scaffolds and delivery systems capable of integrating multiple therapeutic components, including stem cells, growth factors, imaging agents, and biomaterials, into a single platform for synergistic regenerative therapy^[21]^. These multifunctional systems can be tailored to address specific pathophysiological features of CVDs, such as ischemia, inflammation, fibrosis, or arrhythmias, by providing spatial and temporal control over therapeutic interventions^[22]^.

## Clinical applications of gene and stem cell therapies in cardiology

Gene therapy involves the delivery of therapeutic genes to target cells or tissues to modulate gene expression, correct genetic defects, or enhance biological functions. In cardiology, gene therapy holds the potential for treating a wide range of cardiovascular disorders, including ischemic heart disease, heart failure, arrhythmias, and genetic cardiomyopathies^[1]^. Several clinical trials have investigated the safety and efficacy of gene therapy approaches in patients with CVDs, with promising results in some cases. For example, in a landmark clinical trial conducted in patients with severe coronary artery disease, intracoronary administration of adenoviral vectors encoding VEGF was shown to improve myocardial perfusion, reduce angina frequency, and enhance exercise tolerance compared to placebo^[2]^. Similarly, gene therapy targeting sarcoplasmic reticulum calcium ATPase (SERCA2a) has been evaluated in patients with heart failure with reduced ejection fraction (HFrEF), demonstrating improvements in left ventricular function, exercise capacity, and clinical outcomes in some studies^[3]^. Moreover, gene editing technologies, such as CRISPR/Cas9, offer exciting opportunities for precise and targeted manipulation of the cardiac genome, enabling the correction of genetic mutations associated with inherited cardiomyopathies, such as hypertrophic cardiomyopathy and dilated cardiomyopathy^[4]^. Clinical trials evaluating the safety and efficacy of CRISPR-based gene editing therapies in patients with monogenic forms of cardiomyopathy are currently underway, with preliminary results showing promise for potentially correcting disease-causing mutations and restoring cardiac function^[5]^. Stem cell therapy involves transplanting stem cells or progenitor cells into the heart to promote tissue repair, neovascularization, and functional recovery following myocardial injury. Various types of stem cells, including mesenchymal stem cells (MSCs), cardiac progenitor cells (CPCs), and induced pluripotent stem cells (iPSCs), have been investigated for their therapeutic potential in cardiovascular regeneration^[6]^. Clinical trials evaluating the safety and efficacy of stem cell therapies in patients with AMI, chronic heart failure, and other cardiovascular conditions have yielded mixed results. Some studies have shown intracoronary infusion of bone marrow-derived MSCs in patients with AMI to improve left ventricular function, reduce infarct size, and prevent adverse remodeling^[7]^. However, the clinical benefits of stem cell therapy in AMI remain controversial, with other trials reporting no significant improvement in clinical outcomes compared to standard care^[8]^. Similarly, intramyocardial injection of autologous CPCs or allogeneic cardio-sphere-derived cells (CDCs) has demonstrated safety and feasibility in patients with chronic heart failure. Still, it has yielded inconsistent results regarding cardiac function improvement and clinical endpoints^[9]^. Despite these challenges, ongoing research efforts are focused on optimizing stem cell delivery methods, cell types, dosages, and patient selection criteria to enhance the therapeutic efficacy of stem cell therapies in cardiology^[10]^. Novel strategies, such as genetic modification of stem cells to enhance their survival, retention, and paracrine signaling, as well as the use of tissue engineering approaches to create bioactive scaffolds and microenvironments, are being explored to improve the engraftment and functional integration of transplanted cells in the heart^[11]^. Several key research areas and prospects hold promise for advancing gene and stem cell therapies in cardiology and addressing current limitations and challenges. One such area is the development of novel delivery platforms and technologies for targeted and efficient delivery of therapeutic genes and cells to the heart^[12]^. Nanoparticle-based carriers, biomimetic scaffolds, and tissue engineering approaches offer innovative solutions for enhancing gene and stem cell therapies’ specificity, safety, and therapeutic efficacy in cardiovascular regeneration^[16]^. Moreover, integrating gene and stem cell therapies with other therapeutic modalities, such as pharmacological agents, cardiac devices, and regenerative medicine approaches, holds the potential for synergistic and personalized treatment strategies tailored to individual patient needs^[13]^. Combinatorial therapies targeting multiple pathophysiological pathways implicated in CVDs, such as inflammation, fibrosis, and oxidative stress, may offer superior outcomes compared to monotherapy approaches and address the heterogeneity of cardiovascular conditions^[14]^. Furthermore, advances in molecular imaging and noninvasive monitoring techniques enable real-time visualization and tracking of therapeutic genes and cells in vivo, providing valuable insights into their biodistribution, survival, and therapeutic effects^[15]^. Multimodal imaging platforms, including MRI, PET, and bioluminescence imaging (BLI), offer complementary information and enable longitudinal assessment of gene and stem cell therapies in preclinical and clinical settings, facilitating the optimization of treatment protocols and translation to clinical practice^[17]^. Additionally, the development of patient-specific and disease-specific models, including iPSC-derived cardiomyocytes and organoid systems, enables personalized screening of therapeutic interventions and drug discovery in vitro, minimizing the reliance on animal models and accelerating the pace of translational research^[18]^. These advanced models recapitulate key aspects of human cardiac physiology and pathology, providing valuable platforms for studying disease mechanisms, screening candidate drugs, and optimizing treatment strategies for individual patients^[19]^. Furthermore, ongoing efforts to address key challenges and limitations, such as immune rejection, tumorigenicity, and off-target effects associated with gene and stem cell therapies, are critical for ensuring their safety, efficacy, and clinical translation^[20]^. Novel gene editing technologies, including base editing and prime editing, offer precise and efficient methods for correcting disease-causing mutations and modulating gene expression without introducing double-strand breaks or foreign DNA, minimizing the risk of adverse effects^[21]^. Similarly, advances in stem cell engineering, including genome editing, synthetic biology, and cell fate modulation, enable the generation of safer, more stable, and functionally superior cell therapies for cardiovascular regeneration^[22]^. Moreover, the integration of regenerative therapies with emerging technologies, such as AI, ML, and big data analytics, holds promise for advancing precision medicine and optimizing treatment outcomes in cardiology^[23]^. AI-driven algorithms can analyze large-scale omics data, clinical records, and imaging studies to identify predictive biomarkers, stratify patient populations, and optimize treatment protocols, enabling personalized and data-driven decision-making in clinical practice^[24]^.

## Real-time health monitoring

One of the most commonly employed nanosensors in wearable devices is the electrochemical sensor, which detects changes in electrical signals or currents resulting from biochemical reactions. These sensors utilize nanomaterials such as CNT, graphene, and metallic nanoparticles to enhance sensitivity, selectivity, and stability^[71]^. For example, glucose biosensors based on nanomaterial-modified electrodes enable continuous monitoring of blood glucose levels, providing valuable information for diabetic management and risk stratification of cardiovascular complications^[72]^. Similarly, wearable electrochemical sensors for detecting cardiac biomarkers, such as troponin, brain natriuretic peptide (BNP), and C-reactive protein (CRP), offer early warning signs of MI, heart failure, and inflammation, facilitating timely interventions and improving patient outcomes^[73]^. Another important category of nanosensors in wearable devices is optical sensors, which utilize light-matter interactions to detect changes in optical properties such as absorbance, fluorescence, and refractive index. These sensors often employ nanomaterials such as QDs, plasmonic nanoparticles, and fluorescent nanoprobes to enhance sensitivity and specificity^[74]^. For instance, wearable optical sensors based on gold nanoparticles or QDs can detect changes in blood oxygenation levels, providing valuable insights into cardiac function and tissue perfusion^[75]^. Moreover, fluorescent nanoprobes conjugated with specific ligands or antibodies enable targeted detection of circulating biomarkers or molecular targets associated with CVDs, offering high sensitivity and multiplexing capabilities^[76]^. In addition to electrochemical and optical sensors, nanotechnology has enabled the development of other types of nanosensors with unique capabilities for wearable devices. For example, piezoelectric nanosensors detect mechanical deformations or vibrations resulting from physiological activities such as heartbeats or breathing, offering a label-free and energy-efficient approach to monitoring cardiovascular parameters^[77]^. Similarly, magnetic nanosensors exploit changes in magnetic properties such as magnetization or susceptibility to detect biomolecular interactions or changes in tissue properties, providing insights into cardiovascular function and pathology^[78]^. Furthermore, acoustic nanosensors utilize ultrasound waves or surface acoustic waves to detect changes in tissue density, stiffness, or motion, offering noninvasive and high-resolution imaging of cardiovascular structures and functions^[79]^. Integrating nanosensors into wearable devices enables continuous monitoring of cardiovascular parameters in real-time, empowering individuals to track their health status and take proactive measures to prevent or manage CVDs. These wearable devices offer several advantages over traditional monitoring techniques, including portability, convenience, and accessibility, making them ideal for remote patient monitoring, home healthcare, and telemedicine applications^[10]^. Moreover, nanosensors’ miniaturization and low-power consumption enable long-term monitoring without frequent battery replacement or recharging, enhancing user compliance and acceptance^[11]^. Implantable nanosensors utilize nanotechnology-based materials and sensing mechanisms to detect and quantify specific biomarkers or physiological parameters within the body. These sensors are typically fabricated using biocompatible materials such as polymers, ceramics, or metals, which ensure minimal tissue reaction and long-term stability within the biological environment^[1]^. The sensing elements of implantable nanosensors are designed to interact with target molecules or analytes, producing measurable signals such as electrical, optical, or mechanical changes in response to their presence or concentration^[2]^. For example, electrochemical nanosensors employ nanomaterial-modified electrodes to detect changes in electrical currents resulting from biochemical reactions. In contrast, optical nanosensors utilize light-matter interactions to measure changes in optical properties such as absorbance, fluorescence, or refractive index^[3]^. One of the key applications of implantable nanosensors in cardiology is the real-time monitoring of cardiac biomarkers for the early detection and management of CVDs. These biomarkers, which include proteins, enzymes, hormones, and genetic markers, provide valuable insights into cardiac function, tissue damage, and disease progression, enabling timely interventions and personalized treatment strategies^[4]^. For instance, implantable nanosensors can continuously monitor biomarkers such as troponin, brain natriuretic peptide (BNP), and cardiac troponin I (cTnI), which are indicative of MI, heart failure, and other cardiac conditions^[5]^. By providing real-time data on biomarker levels, implantable nanosensors enable healthcare providers to detect cardiac events early, assess disease severity, and adjust treatment plans accordingly, leading to improved patient outcomes and reduced healthcare costs^[6]^. Implantable nanosensors offer several benefits for patient management in cardiology, particularly in chronic diseases such as heart failure, arrhythmias, and hypertension. One of the primary advantages is the ability to continuously monitor biomarkers and physiological parameters, eliminating the need for periodic blood tests or clinic visits^[7]^. This continuous monitoring allows for early detection of changes in cardiac function or disease progression, enabling timely interventions and preventing adverse events such as heart attacks or strokes^[80]^. Moreover, implantable nanosensors can facilitate remote monitoring of patients, allowing healthcare providers to monitor their condition in real time and intervene as needed, regardless of their location^[9]^. This remote monitoring capability improves patient convenience, reduces healthcare costs, and enhances patient satisfaction with their care^[10]^. Furthermore, implantable nanosensors offer unparalleled sensitivity and specificity for detecting biomarkers, enabling early diagnosis of CVDs and personalized treatment strategies. These sensors can detect biomarker levels within the physiological range, providing accurate measurements even in low concentrations or interfering substances^[11]^. This high sensitivity detects subtle changes in cardiac function or disease progression, which may not be apparent with traditional diagnostic methods^[12]^. Additionally, implantable nanosensors can be customized to target specific biomarkers or disease pathways, allowing for personalized monitoring and treatment optimization based on individual patient characteristics^[16]^. This personalized approach to patient management improves treatment outcomes, reduces adverse events, and enhances patient quality of life^[13]^. Implantable nanosensors provide continuous monitoring of various physiological parameters and biomarkers. When integrated with telemedicine platforms, these nanosensors can transmit real-time health data to healthcare providers, allowing for timely and accurate assessment of a patient’s condition without needing physical visits to healthcare facilities^[1]^. This capability is particularly advantageous for patients with chronic CVDs who require frequent monitoring of parameters such as heart rate, blood pressure, glucose levels, and specific cardiac biomarkers like troponin and brain natriuretic peptide (BNP)^[2]^. Real-time data transmission enables early detection of adverse events or exacerbations, allowing for prompt medical intervention to prevent hospitalizations and reduce morbidity and mortality^[3]^. The integration of nanosensors with telemedicine relies on sophisticated data transmission technologies. These systems typically use wireless communication protocols like Bluetooth, Wi-Fi, or cellular networks to transmit data from the nanosensors to a central server or healthcare providers^[4]^. Advances in cloud computing and data analytics play a critical role in managing and interpreting the vast amounts of data generated by nanosensors. Cloud-based platforms can store and analyze patient data, providing healthcare providers with actionable insights and predictive analytics to guide treatment decisions^[5]^. Moreover, AI and ML algorithms can detect data patterns and anomalies, enhancing remote monitoring accuracy and efficiency^[6]^. One of the significant impacts of integrating nanosensors with telemedicine is the potential to improve healthcare accessibility, particularly for patients in remote or underserved areas. Many rural and low-income populations face barriers to accessing quality healthcare due to geographic, economic, and logistical constraints^[7]^. Telemedicine can bridge these gaps by providing remote access to specialized care and continuous monitoring. Patients can receive expert consultations, follow-up care, and monitoring from the comfort of their homes, reducing the need for travel and the associated costs^[8]^. This improved accessibility can lead to earlier diagnosis and treatment, better disease management, and, ultimately, better health outcomes for these populations^[9]^. Additionally, telemedicine combined with nanosensor technology can enhance patient engagement and self-management of chronic diseases. Patients equipped with wearable or implantable nanosensors can receive real-time feedback on their health status, empowering them to make informed decisions about their lifestyle and treatment adherence^[10]^. For example, a patient with heart failure can monitor their fluid status and make dietary or medication adjustments based on the data transmitted by the nanosensors. This active involvement in their care can lead to improved treatment adherence, better management of symptoms, and a greater sense of control over their health^[11]^. Furthermore, integrating nanosensors and telemedicine can optimize healthcare resources and reduce the burden on healthcare systems. Continuous remote monitoring can decrease the frequency of hospital visits and admissions, easing the strain on healthcare facilities and allowing healthcare providers to allocate their resources more efficiently^[12]^. Early detection and intervention facilitated by real-time monitoring can prevent complications and reduce the need for more intensive and costly treatments, leading to significant cost savings for healthcare systems^[16]^. In addition, remote monitoring can help healthcare providers manage larger patient populations more effectively, particularly in regions with a shortage of healthcare professionals^[13]^. Despite these benefits, several challenges and considerations are associated with integrating nanosensors and telemedicine. One of the primary concerns is data security and patient privacy. The transmission and storage of sensitive health data require robust cybersecurity measures to protect against data breaches and unauthorized access^[14]^. Ensuring compliance with regulations such as the Health Insurance Portability and Accountability Act (HIPAA) in the United States and the General Data Protection Regulation (GDPR) in Europe is essential to safeguard patient privacy and maintain trust in telemedicine services^[15]^. Another challenge is the technological infrastructure required to support telemedicine and nanosensor integration. Reliable internet connectivity and access to digital devices are prerequisites for effective telemedicine services, which may be lacking in some remote or underserved areas^[17]^. Efforts to improve digital infrastructure and provide affordable access to technology are crucial for the widespread adoption of telemedicine^[18]^. Additionally, healthcare providers need adequate training and support to effectively utilize telemedicine platforms and interpret data from nanosensors^[19]^. Investment in education and training programs is essential to equip healthcare professionals with the necessary skills to leverage these technologies^[20]^. Moreover, regulatory and reimbursement issues need to be addressed to facilitate the integration of telemedicine and nanosensor technology. The regulatory framework for telemedicine varies widely across regions and countries, impacting the ability of healthcare providers to offer remote services^[21]^. Harmonizing regulations and establishing clear guidelines for telemedicine practices can support its broader adoption. Similarly, reimbursement policies for telemedicine services must be standardized to ensure that healthcare providers are adequately compensated for remote consultations and monitoring^[22]^. Aligning reimbursement models with the value provided by telemedicine can incentivize its use and support sustainable healthcare delivery^[23]^.

## Biocompatible implants

Stents commonly treat narrowed or blocked arteries, primarily in coronary artery disease. However, traditional stents are prone to complications such as restenosis, where the artery narrows again due to tissue growth, and thrombosis, where blood clots form on the stent surface^[1]^. Nanocoatings can mitigate these issues by providing a surface that resists tissue proliferation and clot formation. For instance, drug-eluting stents (DES) have a polymer coating that releases antiproliferative drugs over time, reducing the risk of restenosis^[2]^. Incorporating nanotechnology enhances this approach by allowing for more precise control over drug release and coating properties. One of the significant advantages of nanocoatings is their ability to provide a more uniform and controlled release of therapeutic agents. Nanoparticles can be engineered to release drugs at a specific rate tailored to the patient’s needs^[3]^. This targeted drug delivery can significantly reduce restenosis rates. For example, stents coated with nanoparticles containing sirolimus or paclitaxel have shown improved efficacy in preventing restenosis compared to traditional stents^[4]^. Additionally, nanocoatings can incorporate multiple drugs, simultaneously addressing different pathways involved in restenosis and thrombosis^[5]^. Thrombosis remains a major concern with stent implantation. Nanocoatings can enhance the hemocompatibility of stents, reducing the risk of clot formation. One approach involves using heparin or other anticoagulant agents embedded in nanocoatings to provide a localized, long-term antithrombotic effect^[6]^. Research has shown that stents coated with heparin-loaded nanoparticles exhibit significantly lower thrombogenicity than bare-metal stents^[7]^. Another strategy employs bioinspired nanocoatings that mimic the natural endothelial layer, promoting endothelial cell growth while inhibiting platelet adhesion and activation^[8]^. Valves used in heart valve replacement or repair also benefit from nanocoatings. Traditional valve prostheses, whether mechanical or bioprosthetic, face challenges such as calcification, thrombosis, and limited durability^[9]^. Nanocoatings can enhance the biocompatibility and mechanical properties of these valves. For example, valves coated with diamond-like carbon (DLC) or titanium nitride (TiN) nanocoatings exhibit improved wear resistance and reduced thrombogenicity^[10]^. These coatings provide a smooth, inert surface that minimizes the interaction with blood components, reducing the risk of clot formation and calcification^[11]^. Moreover, nanocoatings can be designed to promote the integration of the valve with the surrounding tissue, enhancing its biocompatibility and reducing the immune response. For instance, coatings incorporating bioactive molecules such as VEGF or nitric oxide (NO) can promote endothelialization and inhibit inflammatory responses^[12]^. Studies have demonstrated that valves coated with NO-releasing nanoparticles exhibit enhanced endothelial cell coverage and reduced inflammation, leading to better long-term outcomes^[16]^. The durability of stents and valves is another critical factor that nanocoatings can improve. Traditional coatings may wear off or degrade over time, compromising the device’s performance. Nanocoatings, however, offer superior mechanical properties, including increased hardness and resistance to wear and corrosion^[13]^. This increased durability can extend the lifespan of stents and valves, reducing the need for repeat interventions^[14]^. For example, stents coated with DLC or TiN nanocoatings have demonstrated excellent long-term stability and resistance to mechanical stress^[15]^. Despite the promising potential of nanocoatings, there are challenges to their widespread adoption. One of the primary concerns is the long-term biocompatibility and safety of nanomaterials used in medical devices. While many studies have shown positive results, the long-term effects of nanoparticles in the body are not yet fully understood, necessitating extensive preclinical and clinical testing^[17]^. Regulatory approval processes for nanocoated devices are also complex, requiring comprehensive evaluation of their safety and efficacy^[18]^. Manufacturing and scalability are other challenges. The production of uniform and consistent nanocoatings requires advanced fabrication techniques and stringent quality control measures. Ensuring reproducibility and scalability while maintaining the desired properties of the nanocoatings can be technically demanding and costly^[19]^. Moreover, integrating nanotechnology into existing manufacturing processes for stents and valves must be seamless to facilitate adoption by the medical device industry^[20]^. Nanocomposites are designed by incorporating nanoscale fillers like CNT, graphene, or nanoparticles into a matrix material, typically a polymer, ceramic, or metal. These nanoscale fillers impart unique properties not achievable with conventional materials alone. For instance, CNTs and graphene can significantly enhance the composite material’s mechanical strength, electrical conductivity, and thermal stability^[1]^. For example, the high aspect ratio and exceptional mechanical properties of CNTs make them ideal for reinforcing polymers, resulting in composites that exhibit increased tensile strength and modulus^[2]^. In the context of orthopedic implants, nanocomposites offer improved mechanical properties essential for mimicking the natural bone’s strength and flexibility. Traditional materials like titanium alloys, stainless steel, and cobalt-chromium alloys have been widely used for bone implants. Still, they often fall short of matching the bone’s mechanical properties and can lead to stress shielding, where the implant takes on too much load, causing bone resorption around it^[3]^. Nanocomposites, particularly those reinforced with hydroxyapatite (HA) nanoparticles, solve this problem. HA is a naturally occurring mineral in bone, and its inclusion in a polymer matrix can improve the composite’s biocompatibility, osteoconductivity, and mechanical strength, making it more similar to natural bone^[4]^. One notable example is HA nanoparticle-reinforced polyetheretherketone (PEEK) composites. PEEK is a high-performance polymer with excellent mechanical properties and chemical resistance. When reinforced with HA nanoparticles, PEEK composites exhibit enhanced mechanical properties and biocompatibility, making them suitable for load-bearing orthopedic implants such as spinal cages and joint replacements^[5]^. These nanocomposites match the mechanical properties of bone more closely and promote better integration with the surrounding bone tissue, leading to improved long-term outcomes^[6]^. In dental applications, nanocomposites have shown significant potential in improving the performance of restorative materials. Dental composites traditionally consist of a resin matrix reinforced with micron-sized filler particles. However, these materials often have inadequate mechanical properties, leading to wear and failure. Incorporating nanoscale fillers, such as silica nanoparticles, into the resin matrix can enhance the composite’s mechanical properties, including increased hardness, wear resistance, and fracture toughness^[7]^. These improvements extend the lifespan of dental restorations and reduce the frequency of replacements, benefiting both patients and dental practitioners. Cardiovascular implants, such as stents and heart valves, also benefit from the development of nanocomposite materials. Traditional stents are made from metals like stainless steel or cobalt-chromium alloys, which can cause complications such as in-stent restenosis (ISR) and thrombosis^[8]^. Nanocomposites can address these issues by enhancing stents’ surface properties and mechanical performance. For example, stents coated with nanocomposites that incorporate drug-eluting nanoparticles or bioactive agents can reduce ISR by releasing therapeutic agents over time^[9]^. Additionally, using nanocomposites with improved mechanical properties ensures that the stents maintain their structural integrity under physiological conditions, reducing the risk of fracture or deformation^[10]^. One promising area of research involves using graphene-based nanocomposites for cardiovascular applications. Graphene’s exceptional electrical conductivity, mechanical strength, and biocompatibility make it an ideal candidate for enhancing stent performance. Graphene-coated stents have demonstrated reduced platelet adhesion and improved endothelial cell proliferation, which are critical factors in preventing thrombosis and promoting vascular healing^[11]^. Moreover, graphene-based nanocomposites can be engineered to release controlled drugs, further enhancing their therapeutic potential^[12]^. Future implants may leverage advanced nanocomposite materials to achieve unprecedented performance and functionality. One emerging trend is the development of smart nanocomposites that can respond to physiological changes or external stimuli. For example, shape-memory polymers reinforced with nanoscale fillers can be designed to change shape in response to temperature changes. This allows for minimally invasive implantation and precise fitting to the target tissue^[16]^. These smart materials have the potential to revolutionize the design and functionality of implants, offering personalized and adaptive solutions for various medical conditions. Another promising avenue is the use of biodegradable nanocomposites for temporary implants. Traditional permanent implants can pose long-term complications, including infection, inflammation, and the need for revision surgery. Biodegradable nanocomposites, made from polymers such as PLA reinforced with nanoscale fillers, can provide the necessary mechanical support during the healing process and then degrade safely within the body^[13]^. These materials are particularly advantageous for pediatric applications, where the growing body requires temporary support that can be gradually resorbed^[14]^. In tissue engineering, nanocomposites are crucial in developing scaffolds for regenerating damaged tissues. These scaffolds must mimic the ECM’s mechanical and biological properties to support cell growth and differentiation. Nanocomposites incorporating bioactive nanoparticles, such as silica or calcium phosphate, into a biodegradable polymer matrix can create scaffolds with enhanced mechanical strength, bioactivity, and biocompatibility^[15]^. These scaffolds provide a conducive environment for tissue regeneration, promoting the formation of new bone, cartilage, or other tissues^[17]^. Despite the promising potential of nanocomposites, several challenges remain in their development and application. One primary concern is the potential toxicity of nanoscale fillers. While many studies have demonstrated the biocompatibility of certain nanomaterials, such as HA or silica nanoparticles, other materials like CNT and graphene can pose toxicity risks depending on their size, shape, and surface properties^[18]^. Comprehensive biocompatibility and toxicity assessments are essential to ensure the safety of nanocomposite implants. Manufacturing and scalability are also significant challenges. Producing nanocomposites with uniform dispersion of nanoscale fillers and consistent properties can be technically demanding and costly. Advanced fabrication techniques, such as electrospinning, 3D printing, and additive manufacturing, are being explored to address these challenges^[19]^. These techniques offer precise control over the material’s microstructure and composition, enabling the production of complex, patient-specific implants with tailored properties^[20]^. Biocompatibility refers to a material’s ability to perform its desired function without eliciting undesirable local or systemic effects in the host^[1]^. In medical implants, biocompatibility is paramount as it ensures that the implant can integrate seamlessly with the surrounding biological tissues. This integration minimizes the risk of inflammation, infection, and rejection, common complications associated with non-biocompatible materials. One of the most significant clinical applications of biocompatible implants is in orthopedics, where they are used extensively for joint replacements, fracture fixation, and spinal surgery. For instance, titanium and its alloys have become a standard in orthopedic implants due to their excellent biocompatibility, mechanical strength, and corrosion resistance^[2]^. Titanium implants promote osseointegration, the process by which the implant bonds directly to bone, providing stability and longevity^[3]^. A case study involving a 65-year-old patient with severe osteoarthritis in the hip provides a clear example of the benefits of biocompatible implants. The patient underwent a total hip replacement using a titanium alloy implant. Postoperative outcomes were favorable, with the patient experiencing significant pain relief and improved mobility. Radiographic analysis showed excellent osseointegration, with no signs of implant loosening or adverse tissue reactions^[4]^. This case underscores the effectiveness of biocompatible materials in enhancing patient outcomes and quality of life. Dental implants are another area where biocompatible materials have made a profound impact. Dental implants, typically made of titanium or zirconia, replace missing teeth and provide a stable foundation for prosthetic teeth. The biocompatibility of these materials ensures that they integrate well with the jawbone, reducing the risk of implant failure and promoting long-term success^[5]^. A study involving 100 patients who received titanium dental implants reported a high success rate, with over 95% of implants remaining stable and functional after 5 years. The study highlighted the importance of biocompatibility in achieving favorable outcomes in dental implantology^[6]^. Cardiovascular implants, such as stents and heart valves, benefit significantly from biocompatible materials. These implants must function effectively within the dynamic environment of the cardiovascular system, where they are exposed to constant blood flow and pressure. Biocompatible materials such as nitinol, a nickel-titanium alloy, are commonly used for stents due to their excellent flexibility, biocompatibility, and ability to transform shape memory^[7]^. A clinical trial involving patients with coronary artery disease who received nitinol stents demonstrated a marked reduction in restenosis rates compared to traditional stainless-steel stents. Patients showed improved arterial patency and fewer adverse cardiovascular events over a 12-month follow-up period^[8]^. Heart valve replacements have also seen significant advancements with biocompatible materials. Mechanical heart valves, traditionally made from metal alloys, have been associated with complications such as thrombosis, necessitating lifelong anticoagulation therapy. In contrast, biocompatible polymeric and bioprosthetic valves made from animal tissues (e.g., bovine or porcine) have shown improved biocompatibility, reducing the risk of thromboembolic events and the need for anticoagulation^[9]^. A study of patients undergoing aortic valve replacement with bioprosthetic valves reported improved hemodynamic performance and lower incidence of valve-related complications, leading to better patient outcomes and quality of life^[10]^. The potential for future innovations in biocompatible implants is vast, with ongoing research focused on developing materials that integrate well with biological tissues and promote healing and tissue regeneration. One exciting area of innovation is the development of smart implants that can respond to changes in the physiological environment. For example, implants coated with bioactive molecules such as growth factors or anti-inflammatory agents can promote tissue healing and reduce the risk of infection^[11]^. These smart coatings can be engineered to release therapeutic agents in a controlled manner, enhancing the healing process and improving long-term outcomes. Nanotechnology is another frontier in the development of biocompatible implants. Nanomaterials offer unique properties, including high surface area-to-volume ratios and the ability to interact at the cellular and molecular levels. These properties make nanomaterials ideal for enhancing the biocompatibility and functionality of implants^[12]^. For instance, nano-HA coatings on titanium implants have promoted bone cell adhesion and proliferation, accelerating osseointegration^[16]^. Similarly, nanoparticles loaded with antibiotics can be incorporated into implant surfaces to provide localized, sustained antimicrobial activity, reducing the risk of postoperative infections^[13]^. Stem cell technology also holds promise for the future of biocompatible implants. Combining stem cells with biocompatible scaffolds can create regenerative implants capable of repairing or replacing damaged tissues. For example, scaffolds made from biocompatible polymers such as PLGA can be seeded with stem cells to create constructs for bone and cartilage regeneration^[14]^. Clinical trials using stem cell-seeded scaffolds for repairing critical-sized bone defects have shown encouraging results, with patients demonstrating significant bone regeneration and functional recovery^[15]^. Integrating 3D printing technology with biocompatible materials is revolutionizing the fabrication of custom implants tailored to individual patients’ specific anatomical and functional requirements. 3D printing allows for precise control over the design and composition of implants, enabling the production of complex structures that closely mimic natural tissues^[17]^. For example, patient-specific cranial implants made from biocompatible polymers have been successfully used to reconstruct skull defects, providing excellent aesthetic and functional outcomes^[18]^. Similarly, 3D-printed orthopedic implants have been designed to match the unique geometry of bone defects, improving implant fit and stability^[19]^. Despite the promising advancements, challenges remain in biocompatible implants’ development and clinical implementation. Ensuring the long-term biocompatibility and safety of new materials is paramount. Rigorous preclinical and clinical testing is required to evaluate potential adverse reactions, such as chronic inflammation, immunogenicity, and toxicity^[20]^. Regulatory approval processes must be stringent and comprehensive to ensure that new biocompatible materials meet the highest standards of safety and efficacy^[21]^. Additionally, the scalability and cost-effectiveness of manufacturing biocompatible implants pose significant challenges. Advanced fabrication techniques like nanotechnology and 3D printing can be expensive and technically demanding. Developing scalable and cost-effective manufacturing processes is crucial to making biocompatible implants accessible to a broader patient population^[22]^. Collaborations between researchers, industry, and regulatory bodies will be essential to address these challenges and accelerate the translation of innovative biocompatible materials into clinical practice.

## Regenerative medicine

The primary advantage of using nanoparticles in tissue engineering lies in their ability to deliver high-precision and efficacy therapeutic agents. Growth factors and cytokines are critical signaling molecules that regulate cell behavior, tissue growth, and healing processes. However, their clinical application is often limited by their rapid degradation, short half-life, and potential for systemic side effects when administered in large doses^[1]^. Nanoparticles can address these challenges by providing a controlled and sustained release of these bioactive molecules, enhancing their therapeutic effects, and minimizing adverse reactions. One of the most extensively studied types of nanoparticles for growth factor delivery is the polymeric nanoparticle. These nanoparticles are typically composed of biodegradable polymers such as PLGA, which can encapsulate growth factors and release them in a controlled manner as the polymer degrades^[2]^. For example, PLGA nanoparticles loaded with VEGF have been shown to promote angiogenesis and the formation of new blood vessels, which is crucial for tissue regeneration and healing^[3]^. In a study involving a rat model of MI, the delivery of VEGF via PLGA nanoparticles resulted in significantly improved cardiac function and reduced scar tissue formation compared to controls^[4]^. This demonstrates the potential of nanoparticle-mediated growth factor delivery in enhancing cardiac tissue regeneration. Another promising approach involves using lipid-based nanoparticles, such as liposomes, which can encapsulate hydrophilic and hydrophobic molecules, making them versatile carriers for a wide range of growth factors and cytokines^[5]^. Liposomes can be engineered to release their payload in response to specific environmental triggers, such as pH changes or enzymatic activity, providing targeted and controlled release^[6]^. For instance, liposomes loaded with transforming growth factor-beta (TGF-β) have been used to promote the differentiation of stem cells into cardiomyocytes. These cells make up heart muscle tissue^[7]^. This targeted delivery approach enhances the regenerative potential of stem cell therapies, offering a promising strategy for repairing damaged cardiac tissue. In addition to delivering growth factors, nanoparticles can serve as scaffolds for tissue engineering. Scaffolds provide a three-dimensional structure that supports cell attachment, proliferation, and differentiation, guiding the formation of new tissue^[8]^. Nanoparticles can be incorporated into these scaffolds to enhance their mechanical properties, bioactivity, and functionality. For cardiac tissue regeneration, developing scaffolds that mimic the heart’s natural ECM is crucial for supporting the growth and function of cardiac cells. Electrospun nanofibers are a popular type of scaffold in tissue engineering due to their high surface area-to-volume ratio and ability to mimic the fibrous structure of natural ECM^[9]^. Incorporating nanoparticles into electrospun nanofibers can enhance their mechanical strength and bioactivity. For example, scaffolds made from electrospun nanofibers containing gold nanoparticles have been shown to improve the electrical conductivity of the scaffold, which is essential for the synchronized contraction of cardiac cells^[10]^. In a study using a rat model of MI, cardiac patches made from these gold nanoparticle-incorporated nanofibers significantly improved cardiac function and reduced arrhythmias compared to patches without nanoparticles^[11]^. Another innovative approach involves using hydrogels, which are water-swollen, crosslinked polymer networks that can provide a highly hydrated environment similar to natural tissues. Nanoparticles can be embedded within hydrogels to enhance their mechanical properties and deliver bioactive molecules. For example, hydrogels containing silica nanoparticles have been used to deliver IGF-1, a key molecule in cardiac tissue repair and regeneration^[12]^. In a study involving a porcine model of MI, IGF-1-loaded silica nanoparticle hydrogels improved cardiac function and reduced scar tissue formation compared to controls^[16]^. This highlights the potential of nanoparticle-enhanced hydrogels in cardiac tissue engineering. Moreover, nanoparticles can be designed to provide multiple therapeutic functions simultaneously. For instance, multifunctional nanoparticles can be engineered to deliver both growth factors and genes that promote tissue regeneration. Such an approach can synergistically enhance the regenerative process by providing the cells with the necessary biochemical signals and genetic instructions^[13]^. In a study using a mouse model of MI, nanoparticles co-delivering VEGF and a gene encoding for stromal cell-derived factor-1 (SDF-1) showed superior cardiac repair and regeneration compared to nanoparticles delivering either agent alone^[14]^. This multifunctional approach demonstrates the potential of nanoparticles to provide comprehensive and effective treatment strategies for cardiac tissue regeneration. Future innovations in nanoparticle-enhanced tissue engineering will likely focus on developing more sophisticated delivery systems and integrating advanced materials. For example, using stimuli-responsive nanoparticles that release their payload in response to specific biological cues, such as inflammation or hypoxia, could provide more precise and dynamic control over the delivery of therapeutic agents^[15]^. Additionally, incorporating nanomaterials such as graphene and CNT into scaffolds could enhance their mechanical properties and electrical conductivity, making them more suitable for cardiac tissue engineering^[17]^. Furthermore, advances in nanotechnology and materials science could enable the development of personalized tissue engineering strategies. By tailoring nanoparticles and scaffolds to the specific needs of individual patients, it may be possible to create customized treatments that optimize the regenerative process and improve clinical outcomes^[18]^. This personalized approach could involve patient-specific cells and growth factors and the design of scaffolds that match the target tissue’s unique anatomical and functional requirements. The clinical translation of nanoparticle-enhanced tissue engineering will require rigorous testing and validation to ensure safety and efficacy. This involves preclinical studies in animal models and well-designed clinical trials in human patients. Regulatory approval processes must be stringent and comprehensive, addressing potential concerns related to the long-term biocompatibility and safety of nanoparticles^[19]^. Collaboration between researchers, clinicians, and regulatory agencies will be essential to navigate these challenges and bring these innovative therapies to the clinic. MI, commonly known as a heart attack, occurs when blood flow to a part of the heart is blocked for a long enough period that part of the heart muscle is damaged or dies. This leads to a cascade of pathological events, including inflammation, scar formation, and adverse remodeling, which can eventually lead to heart failure. Heart failure, characterized by the heart’s inability to pump sufficient blood to meet the body’s needs, is a chronic condition often resulting from the long-term effects of MI. Nanoparticle-enhanced tissue engineering aims to address these challenges by facilitating cardiac repair and regeneration by directly delivering growth factors, cytokines, and other therapeutic agents to the damaged heart tissue. One of the key strategies involves using nanoparticles to deliver VEGF, a potent angiogenic factor that promotes the formation of new blood vessels. Enhancing angiogenesis in the infarcted myocardium can improve blood supply, reduce cell death, and support tissue regeneration. A significant advancement in this area is using polymeric nanoparticles, such as those made from PLGA, to encapsulate and release VEGF in a controlled manner. Studies have demonstrated that PLGA nanoparticles loaded with VEGF can sustain its release over an extended period, promoting prolonged angiogenic activity and improving cardiac function post-MI. For instance, in a study using a rat model of MI, VEGF-loaded PLGA nanoparticles were shown to significantly enhance angiogenesis and reduce infarct size compared to free VEGF administration, leading to improved cardiac function^[1]^. Another promising approach involves using nanoparticles to deliver microRNAs (miRNAs), small non-coding RNAs that regulate gene expression and play critical roles in cardiac repair and regeneration. Specific miRNAs, such as miR-21 and miR-126, have been identified to promote angiogenesis and inhibit apoptosis in the infarcted myocardium. Nanoparticles can be engineered to protect these miRNAs from degradation and facilitate their targeted delivery to cardiac cells. In a preclinical study, nanoparticle-mediated delivery of miR-21 to the infarcted myocardium enhanced angiogenesis, reduced fibrosis, and improved cardiac function^[2]^. Hydrogels incorporated with nanoparticles represent another innovative approach in nanoparticle-enhanced tissue engineering for cardiac repair. Hydrogels provide a biomimetic, three-dimensional matrix supporting cell attachment, proliferation, and differentiation. Hydrogels can offer a controlled release of therapeutic agents and improved mechanical properties when combined with nanoparticles. For example, a hydrogel embedded with VEGF-loaded nanoparticles enhanced cardiac repair in a rat model of MI by promoting angiogenesis and reducing scar tissue formation^[3]^. Stem cell therapy is a highly researched area for cardiac repair, where nanoparticles can play a pivotal role in enhancing the efficacy of stem cell-based treatments. CPC, MPC, and iPSCs have shown potential in regenerating damaged heart tissue. However, the survival and integration of these cells in the harsh post-MI environment remain challenging. Nanoparticles can deliver growth factors and other supportive molecules to improve stem cells’ engraftment, survival, and differentiation in the infarcted myocardium. For example, nanoparticle-mediated delivery of IGF-1 alongside stem cells has enhanced cell survival and promoted cardiac repair in preclinical models^[4]^. In addition to growth factors and miRNAs, nanoparticles can also deliver drugs that modulate the immune response and reduce inflammation, a critical aspect of cardiac repair post-MI. For instance, nanoparticles loaded with anti-inflammatory drugs such as dexamethasone have been used to attenuate the inflammatory response in the infarcted myocardium, thereby reducing tissue damage and promoting healing. A study demonstrated that dexamethasone-loaded nanoparticles significantly reduced inflammation and improved cardiac function in a mouse model of MI^[5]^. Nanoparticle-enhanced scaffolds are another vital component of tissue engineering for cardiac repair. Electrospun nanofibers, which mimic the fibrous structure of the native ECM, can be incorporated with nanoparticles to improve their mechanical properties and bioactivity. For instance, scaffolds made from electrospun nanofibers containing gold nanoparticles have been shown to enhance the electrical conductivity of the scaffold, which is crucial for maintaining the synchronized contraction of cardiac cells. In a rat model of MI, these gold nanoparticle-enhanced scaffolds improved cardiac function and reduced arrhythmias^[6]^. Current research also explores using multifunctional nanoparticles that simultaneously deliver multiple therapeutic agents. These nanoparticles can be engineered to release a combination of growth factors, genes, and drugs in a controlled manner, providing a synergistic effect for cardiac repair. For example, a study demonstrated that nanoparticles co-delivering VEGF and a gene encoding SDF-1 significantly enhanced angiogenesis and cardiac repair compared to nanoparticles delivering either agent alone^[7]^. Recent breakthroughs in nanotechnology have also led to the development of stimuli-responsive nanoparticles that can release their payload in response to specific physiological conditions, such as pH changes or enzymatic activity. These nanoparticles can provide targeted and controlled release of therapeutic agents, improving the efficacy and safety of treatments for cardiac repair. For instance, pH-responsive nanoparticles loaded with VEGF were shown to release VEGF in the acidic environment of the infarcted myocardium, promoting targeted angiogenesis and cardiac repair^[8]^. Integrating advanced materials such as graphene and CNT into nanoparticle-enhanced scaffolds represents another exciting area of research. These materials offer exceptional mechanical properties and electrical conductivity, making them suitable for cardiac tissue engineering. For example, graphene oxide nanoparticles incorporated into scaffolds have been shown to improve the scaffold’s mechanical strength and electrical conductivity, enhancing the survival and function of cardiac cells in vitro^[9]^. Despite these promising advancements, several challenges remain in the clinical translation of nanoparticle-enhanced tissue engineering for cardiac repair. Ensuring nanoparticles’ long-term biocompatibility and safety is paramount, as their interactions with biological tissues and potential toxicity must be thoroughly evaluated. Rigorous preclinical studies and well-designed clinical trials are essential to assess the efficacy and safety of these nanoparticle-based therapies. Additionally, the scalability and cost-effectiveness of manufacturing nanoparticle-enhanced scaffolds and delivery systems pose significant challenges. Advanced fabrication techniques such as nanotechnology and 3D printing, while offering precise control over the design and composition of nanoparticles and scaffolds, can be expensive and technically demanding. Developing scalable and cost-effective manufacturing processes is crucial to making these therapies accessible to a broader patient population. The regulatory landscape for nanoparticle-based therapies is another critical consideration. Regulatory approval processes must be stringent and comprehensive to ensure that new nanoparticle-based materials and treatments meet the highest safety and efficacy standards. Collaboration between researchers, industry, and regulatory agencies will be essential to navigate these challenges and accelerate the translation of nanoparticle-enhanced tissue engineering into clinical practice. One of the primary biological barriers in nanoparticle-enhanced tissue engineering is the body’s immune response. The immune system can recognize nanoparticles as foreign invaders, leading to their rapid clearance from the bloodstream by phagocytic cells such as macrophages and monocytes^[1]^. This immune response can significantly reduce the efficacy of nanoparticle-based therapies by limiting their accumulation at the target site. Strategies to overcome this challenge include modifying the surface of nanoparticles with biocompatible materials such as polyethylene glycol (PEG), which can help evade immune detection and prolong circulation time^[2]^. However, PEGylation and other surface modifications can introduce new challenges, such as altered biodistribution and potential long-term toxicity^[3]^. Another significant biological barrier is the delivery of nanoparticles to the target tissue. Effective delivery requires nanoparticles to navigate complex biological environments, including the ECM, blood vessels, and cellular membranes. The dense and highly organized structure of the ECM can impede the penetration and distribution of nanoparticles, reducing their therapeutic efficacy^[4]^. Researchers are developing nanoparticles with enhanced tissue penetration capabilities to address this issue. For example, nanoparticles functionalized with matrix metalloproteinases (MMPs) can degrade ECM components, facilitating deeper tissue penetration and improved therapeutic outcomes^[5]^. The design and fabrication of nanoparticles also present technical limitations. Achieving precise control over nanoparticle size, shape, and surface properties is crucial for optimizing their biological performance. Small variations in these parameters can significantly impact the nanoparticles’ stability, cellular uptake, and biodistribution^[6]^. Advanced fabrication techniques, such as microfluidics and self-assembly, are being explored to produce nanoparticles with uniform and well-defined characteristics^[7]^. However, scaling up these techniques for large-scale production remains challenging, as maintaining consistency and reproducibility at an industrial scale is complex and costly. Another technical limitation is the controlled release of therapeutic agents from nanoparticles. While nanoparticles can provide sustained and localized delivery of drugs, growth factors, and genes, achieving precise control over the release kinetics is challenging. Factors such as nanoparticle degradation rate, environmental conditions, and the interaction between the therapeutic agent and the nanoparticle matrix can influence the release profile^[8]^. Researchers are exploring various strategies to address this issue, including using stimuli-responsive nanoparticles that release their payload in response to specific triggers such as pH, temperature, or enzymatic activity^[9]^. While these approaches show promise, further research is needed to ensure their stability and reliability in vivo. Biocompatibility and toxicity of nanoparticles are critical concerns that must be thoroughly addressed. The long-term effects of nanoparticles on human health and the environment are not yet fully understood. Potential toxicity can arise from the materials used in nanoparticle fabrication, their degradation products, and the accumulation of nanoparticles in organs and tissues^[10]^. Rigorous preclinical and clinical studies are essential to evaluate the safety of nanoparticle-based therapies. Moreover, developing biodegradable and biocompatible nanoparticles that can be safely cleared from the body after delivering their therapeutic payload is a key area of research^[11]^. Despite these challenges, nanoparticle-enhanced tissue engineering offers numerous future research and innovation opportunities. One promising direction is the development of multifunctional nanoparticles that can deliver multiple therapeutic agents simultaneously. Multifunctional nanoparticles can synergistically enhance the therapeutic outcome by co-encapsulating drugs, growth factors, and genes. For example, nanoparticles co-delivering VEGF and a gene encoding for SDF-1 have significantly enhanced angiogenesis and tissue regeneration compared to nanoparticles delivering either agent alone^[12]^. Another exciting opportunity lies in the integration of advanced materials into nanoparticle design. Materials such as graphene, CNT, and gold nanoparticles offer unique properties that enhance tissue engineering scaffolds’ mechanical strength, electrical conductivity, and bioactivity^[16]^. For instance, graphene oxide nanoparticles have been incorporated into hydrogels to improve their mechanical properties and support the growth and differentiation of stem cells^[13]^. These advanced materials can also be functionalized with bioactive molecules to provide additional therapeutic benefits. Personalized medicine is an emerging field that can greatly benefit from nanoparticle-enhanced tissue engineering. By tailoring nanoparticles and scaffolds to the specific needs of individual patients, personalized treatments can optimize the regenerative process and improve clinical outcomes. This approach could involve using patient-specific cells and growth factors and designing scaffolds that match the target tissue’s unique anatomical and functional requirements^[14]^. Advances in 3D printing and bioprinting technologies facilitate the production of customized scaffolds with precise geometries and material compositions, further enhancing the potential for personalized tissue engineering^[15]^. AI and ML in nanoparticle-enhanced tissue engineering is another promising research direction. AI and ML algorithms can analyze large datasets to identify patterns and predict nanoparticle and scaffolds’ optimal design and formulation. These technologies can also assist in optimizing the delivery and release of therapeutic agents, improving the efficiency and efficacy of nanoparticle-based therapies^[17]^. For example, ML models have been used to predict nanoparticles’ cellular uptake and biodistribution based on their physicochemical properties, enabling the rational design of more effective nanoparticles^[18]^. Another area of future research involves the development of biodegradable and bioresorbable nanoparticles. These nanoparticles can degrade into non-toxic byproducts naturally eliminated from the body, reducing the risk of long-term toxicity and accumulation. Biodegradable polymers such as PLGA and PCL are commonly used in the fabrication of these nanoparticles due to their biocompatibility and tunable degradation rates^[19]^. Further research is needed to develop new biodegradable materials with improved properties and to understand these nanoparticles’ degradation mechanisms and kinetics in vivo. Collaboration between researchers, clinicians, and regulatory agencies is essential to overcome the challenges and advance the field of nanoparticle-enhanced tissue engineering. Interdisciplinary research can facilitate the translation of laboratory findings into clinical applications, ensuring that new therapies are safe, effective, and accessible to patients. Regulatory agencies are crucial in setting guidelines and standards for evaluating and approving nanoparticle-based therapies, ensuring they meet the highest safety and efficacy standards^[20]^.

## Minimally invasive surgeries

Nanorobotics involves nanoscale robots, or nanobots, designed to perform specific tasks within the human body. These nanobots can be engineered to navigate through biological environments, deliver drugs, repair tissues, and perform diagnostic functions with high precision. One of the primary advantages of nanorobots is their ability to access hard-to-reach areas within the body, which is particularly beneficial for minimally invasive surgery (MIS). Minimally invasive techniques aim to reduce the physical trauma associated with traditional open surgeries. MIS can minimize pain, scarring, and recovery by utilizing smaller incisions, advanced imaging technologies, and specialized instruments. Nanorobotics enhances these techniques by providing tools that operate at the molecular or cellular level, offering unparalleled precision and control. One notable application of nanorobotics in minimally invasive surgery is targeted drug delivery. Nanobots can be engineered to carry therapeutic agents directly to diseased cells or tissues, reducing systemic side effects and improving treatment efficacy. For instance, nanorobots loaded with chemotherapeutic drugs can navigate the bloodstream to target cancer cells specifically, sparing healthy tissues and minimizing adverse effects^[1]^. This targeted approach enhances the effectiveness of chemotherapy and allows for higher drug concentrations at the tumor site, potentially improving patient outcomes. In addition to targeted drug delivery, nanorobots can perform mechanical tasks at the cellular level, such as removing or repairing damaged tissues. For example, nanorobots equipped with micro-tools can be programmed to excise malignant cells or repair vascular lesions, offering a highly precise alternative to conventional surgical methods. These capabilities are particularly useful in delicate procedures where traditional instruments may cause collateral damage to surrounding healthy tissues. Another promising area of nanorobotics is in the field of endoscopy. Traditional endoscopic procedures involve inserting a flexible tube with a camera and light source into the body to visualize and treat internal organs. While effective, these procedures can be limited by the size and flexibility of the endoscope. Nanoscale instruments, such as nanoscale endoscopes or nanobots, can overcome these limitations by enhancing maneuverability and resolution. Nanoscale endoscopes, for example, utilize optical fibers or nanowires to achieve high-resolution imaging at the cellular level. These instruments can be inserted into narrow and convoluted anatomical pathways, providing detailed images of tissues that are otherwise difficult to access. This capability is invaluable in diagnosing and treating diseases of the gastrointestinal tract, respiratory system, and vascular system^[2]^. Furthermore, nanoscale endoscopes can be equipped with therapeutic modalities, such as lasers or drug delivery systems, enabling simultaneous diagnosis and treatment. Integrating nanorobotics with imaging technologies, such as MRI and ultrasound, further enhances their precision and control in surgical procedures. By combining real-time imaging with nanorobotic manipulation, surgeons can achieve greater accuracy in targeting and treating diseased tissues. For instance, MRI-guided nanorobots can be directed to specific locations within the body using magnetic fields, allowing for precise navigation and intervention^[3]^. This approach is particularly useful in neurosurgery, where the ability to target and treat brain lesions accurately is critical. One of the most groundbreaking applications of nanorobotics in minimally invasive surgery is treating CVDs. Cardiovascular interventions often require navigating through the complex and delicate vascular system to treat conditions such as atherosclerosis, aneurysms, and heart valve defects. Traditional surgical techniques can be invasive and carry significant risks. Nanorobots offer a minimally invasive alternative by providing tools that can travel through blood vessels to perform precise interventions. For example, magnetically controlled nanorobots can deliver clot-dissolving agents directly to thrombi, reducing the risk of stroke and other complications^[4]^. These nanorobots can also be equipped with micro-scale drills or lasers to remove plaque deposits from arterial walls, restoring blood flow and preventing cardiovascular events. Additionally, nanorobots can repair damaged heart valves or deliver stem cells to promote tissue regeneration, offering new therapeutic options for patients with heart disease. Nanorobotics also has significant implications for cancer treatment beyond targeted drug delivery. One innovative approach involves using nanobots for hyperthermia therapy, where localized heating destroys cancer cells. Nanorobots can be designed to generate heat when exposed to external stimuli, such as magnetic fields or light, allowing for precise control over the treatment area^[5]^. This targeted heating can effectively kill cancer cells while minimizing damage to surrounding healthy tissues, offering a less invasive alternative to traditional radiotherapy. The development of nanoscale instruments for surgical procedures is closely linked to advancements in nanomaterials. Materials such as CNT, graphene, and QDs offer unique properties advantageous for medical applications. CNT, for example, are highly conductive and strong, making them suitable for creating nanoscale surgical tools with exceptional precision and durability. These tools can be used to manipulate cellular structures, excise tumors, or repair tissues at the nanoscale level. Graphene, with its outstanding electrical and mechanical properties, can be used in biosensors and imaging agents, enhancing the capabilities of nanoscale instruments in real-time monitoring and diagnosis during surgical procedures^[6]^. QDs, known for their bright and stable fluorescence, can be used for imaging and tracking nanorobots within the body, providing detailed visualization of their interactions with tissues and aiding in precise surgical interventions^[7]^. Despite the promising potential of nanorobotics and nanoscale instruments, several challenges must be addressed to realize their clinical applications fully. One major challenge is the biocompatibility and potential toxicity of nanomaterials. While many nanomaterials exhibit excellent properties for medical applications, their interactions with biological systems can lead to unforeseen adverse effects. Ensuring nanomaterials’ long-term safety and biocompatibility is crucial for their successful integration into clinical practice^[8]^. Comprehensive preclinical studies and rigorous clinical trials are essential to evaluate the safety and efficacy of these technologies. Another challenge is the precise control and navigation of nanorobots within the human body. Accurate targeting and movement in complex biological environments require sophisticated control systems and real-time feedback mechanisms. Advances in remote control technologies, such as magnetic fields, ultrasound, and light-based guidance systems, are being explored to improve the precision and reliability of nanorobot navigation^[9]^. Additionally, integrating AI and ML algorithms can enhance the autonomous capabilities of nanorobots, allowing them to adapt to dynamic biological conditions and optimize their therapeutic actions^[10]^. The scalability and reproducibility of manufacturing nanorobots and nanoscale instruments also pose significant challenges. Producing these devices with consistent quality and at an industrial scale requires advanced fabrication techniques and stringent quality control measures. Techniques such as microfluidics, lithography, and self-assembly are being investigated to achieve precise and scalable production of nanorobots^[11]^. Collaboration between researchers, engineers, and industry stakeholders is essential to develop cost-effective and scalable manufacturing processes to bring these advanced technologies to the clinical market. Regulatory challenges are another critical aspect to consider. The regulatory pathways for approving nanorobotics and nanoscale instruments are complex and require a comprehensive evaluation of their safety, efficacy, and long-term effects. Regulatory agencies must establish clear guidelines and standards for assessing and approving these technologies to ensure their safe and effective use in clinical practice^[12]^. Collaboration between regulatory bodies, researchers, and industry leaders is crucial to navigating the regulatory landscape and expediting nanorobotic technology translation from the laboratory to the clinic. Future research directions in nanorobotics and nanoscale instruments are vast and hold great promise for advancing minimally invasive techniques. One exciting area of research is the development of biohybrid nanorobots, which combine synthetic materials with biological components. These biohybrid nanorobots can leverage the advantages of both worlds, such as the precision and control of synthetic nanomaterials and the biocompatibility and functionality of biological systems. For example, researchers are exploring using bacteria or cells as carriers for nanorobots, enabling them to navigate complex biological environments and deliver therapeutic agents with high precision^[16]^. Another promising research direction is the integration of nanorobotics with tissue engineering and regenerative medicine. Researchers can create advanced scaffolds and delivery systems for tissue regeneration and repair by combining nanorobots with biomaterials and stem cells. Nanorobots can facilitate the precise delivery of growth factors, genes, and stem cells to damaged tissues, enhancing the regenerative process and improving patient outcomes^[13]^. This approach holds significant potential for treating various conditions, from cardiac and neural injuries to musculoskeletal disorders. The development of smart and responsive nanorobots is another exciting avenue of research. These nanorobots can be engineered to respond to specific physiological cues, such as changes in pH, temperature, or enzyme activity, to perform targeted therapeutic actions. For example, pH-responsive nanorobots can release drugs in the acidic microenvironment of tumors, providing localized treatment and minimizing systemic side effects^[14]^. Similarly, temperature-sensitive nanorobots can generate heat for hyperthermia therapy in response to external stimuli, offering precise control over the treatment area^[15]^. One of the most notable benefits of using nanorobotics in cardiology is reduced recovery times. Minimally invasive nanorobot-assisted procedures typically involve fewer body traumas than conventional surgeries. This less invasive approach allows patients to recover more quickly, often requiring only short hospital stays and enabling a faster return to daily activities. For example, nanorobots used in targeted drug delivery systems can administer therapeutic agents directly to the disease site, minimizing systemic side effects and optimizing the therapeutic efficacy^[2]^. This targeted approach not only enhances treatment outcomes but also reduces the burden of recovery from side effects typically seen with conventional systemic drug administration. Nanoscale instruments, such as those used for imaging and diagnostic purposes, provide unprecedented resolution and specificity. These instruments can detect early signs of CVDs with remarkable accuracy, allowing for timely interventions that can prevent the progression of the disease. Enhanced imaging techniques using nanoparticles, such as QDs and nanoparticle-based contrast agents, enable clearer visualization of cardiac tissues and structures. This precise imaging aids in accurate diagnosis and effective treatment planning, which is crucial for patient outcomes^[3]^. For instance, MRI enhanced with nanoparticle-based contrast agents can reveal detailed cardiac anatomy and pathology, aiding in early detection of conditions like MI or heart failure^[4]^. Several case studies have demonstrated nanorobotics and nanoscale instruments’ clinical applications and benefits in cardiology. In one study, nanorobots delivered chemotherapy drugs directly to heart tumors, significantly reducing the side effects commonly associated with conventional chemotherapy^[5]^. This targeted approach not only preserved healthy cardiac tissue but also improved the overall efficacy of the treatment. Another case involved using nanorobots to clear arterial blockages, showcasing their potential to replace traditional angioplasty and stenting procedures. Patients treated with nanorobots experienced shorter hospital stays and quicker recoveries than those undergoing conventional procedures^[6]^. In addition to their therapeutic applications, nanorobots and nanoscale instruments play a crucial role in regenerative medicine. Nanotechnology can enhance stem cell therapies by facilitating the precise delivery and integration of stem cells into damaged cardiac tissues. Nanoparticle-labeled stem cells can be tracked in real-time, allowing clinicians to monitor their distribution, engraftment, and functional integration^[7]^. This capability ensures that stem cell therapies are administered more effectively, leading to improved regeneration of damaged heart tissues and better patient outcomes. Furthermore, nanotechnology’s role in developing biocompatible implants has significant implications for patient recovery and long-term outcomes. For instance, nanocoatings for stents and heart valves have been shown to reduce the incidence of restenosis and thrombosis. These nanocoatings improve the biocompatibility and durability of implants, leading to fewer complications and longer-lasting results^[8]^. Patients receiving these advanced implants typically experience fewer postoperative complications and a reduced need for additional interventions. Research continues to explore the full potential of nanorobotics and nanoscale instruments in cardiology. Ongoing clinical trials are investigating various applications of these technologies, such as using gold nanoparticles for precision therapy and polymeric nanoparticles for sustained drug release^[9]^. These studies aim to validate the safety and efficacy of nanotechnology-based interventions and pave the way for their broader adoption in clinical practice. Despite the promising advancements, several challenges remain in the clinical implementation of nanorobotics and nanoscale instruments. Ensuring the biocompatibility and long-term safety of nanomaterials is paramount. Researchers must conduct rigorous preclinical and clinical evaluations to fully understand the interactions between nanomaterials and biological systems. The scalability and cost-effectiveness of manufacturing these advanced technologies must be addressed to make them accessible to a broader patient population^[10]^. Another critical aspect is the regulatory landscape for nanotechnology-based medical devices. Regulatory agencies must establish clear guidelines and standards for the approval and use of nanorobotics and nanoscale instruments in clinical practice. This involves comprehensive risk assessments and the development of standardized testing protocols to ensure patient safety^[11]^. Collaborative efforts between researchers, clinicians, industry stakeholders, and regulatory bodies are essential to navigate these regulatory challenges and facilitate the successful translation of nanotechnology innovations from the laboratory to the clinic. One of the most promising developments in nanorobotic surgery is the advancement in the design and functionality of nanorobots. Future nanorobots are expected to be more sophisticated and capable of performing a wider range of tasks with higher precision. These nanorobots could be equipped with advanced sensors and actuators to navigate the human body, identify pathological sites, and perform surgical interventions with minimal damage to surrounding tissues. For instance, nanorobots could be designed to clear arterial blockages, repair damaged tissues, or deliver drugs directly to specific cells, thus enhancing the efficacy of treatments while minimizing side effects^[1]^. Another significant trend is integrating AI and ML with nanorobotic systems. AI and ML algorithms can process and analyze vast amounts of data nanorobots collect in real-time, enabling more accurate diagnosis and decision-making during surgical procedures. For example, AI-driven nanorobots could adapt their behavior based on real-time feedback from their environment, improving their ability to target and treat diseased tissues effectively. This integration of AI and nanorobotics is expected to enhance the precision and efficiency of surgeries, leading to better patient outcomes^[2]^. Moreover, advancements in materials science are expected to play a crucial role in the future of nanorobotic surgery. Developing biocompatible and biodegradable materials for constructing nanorobots will be essential to ensure their safe use in the human body. These materials need to be non-toxic, capable of withstanding the body’s physiological conditions and eventually biodegradable or easily excreted from the body after completing their tasks. Research in this area is ongoing, with scientists exploring nanomaterials such as gold nanoparticles, CNT, and polymeric materials for their potential use in nanorobotics^[3]^. In addition to material advancements, developing more efficient energy sources for powering nanorobots is a critical area of research. Current nanorobots are often limited by their energy sources, which can restrict their operational capabilities and longevity. Future innovations may include the development of nanobatteries or energy-harvesting mechanisms that can draw power from the body’s natural processes, such as glucose metabolism or electromagnetic fields within the body. These advancements would enable nanorobots to operate for extended periods, perform more complex tasks, and reduce the need for external energy sources^[4]^. The future of nanorobotic surgery also involves the development of more sophisticated control and communication systems. Ensuring precise control over nanorobots is essential for their safe and effective use in surgery. Advances in wireless communication technologies, such as 5 G and beyond, could enable real-time, high-speed communication between nanorobots and external controllers, allowing for more precise coordination and control during surgical procedures. Furthermore, developing robust control algorithms that can manage the complex dynamics of nanorobots within the human body is a critical area of ongoing research^[5]^. Despite the promising future trends, several challenges need to be addressed to realize the full potential of nanorobotic surgery. One of the primary challenges is ensuring the safety and efficacy of nanorobots in clinical settings. Rigorous preclinical and clinical testing is required to evaluate nanorobots’ biocompatibility, toxicity, and long-term effects on the human body. This involves developing standardized protocols and testing methodologies to accurately assess the safety and performance of nanorobots^[6]^. Another significant challenge is the scalability and cost-effectiveness of manufacturing nanorobots. Producing nanorobots at a scale sufficient for widespread clinical use requires significant advancements in nanofabrication techniques. These techniques must be optimized to ensure high-quality, reproducible production of nanorobots while keeping costs manageable. Collaboration between academia, industry, and regulatory bodies will be crucial to address these manufacturing challenges and facilitate the translation of nanorobotic technologies from the laboratory to clinical practice^[7]^. Regulatory approval is another critical aspect that needs to be addressed. The regulatory landscape for nanorobotic surgery is still evolving, with regulatory agencies working to establish guidelines and standards for the approval and use of nanorobotic devices. Ensuring that nanorobotic technologies meet the stringent safety, efficacy, and quality standards set by regulatory agencies is essential for their successful clinical implementation. This will require ongoing dialogue and collaboration between researchers, industry stakeholders, and regulatory bodies to develop harmonized guidelines and regulatory pathways for nanorobotic surgery^[8]^.

## Customized medicine

Nanoparticles have emerged as versatile drug delivery, imaging, diagnostics, and therapy platforms due to their unique physicochemical properties and biocompatibility. These nanoscale carriers can be engineered to encapsulate therapeutic agents, such as drugs, genes, or imaging contrast agents, and deliver them to specific targets within the body with enhanced precision and efficacy. By functionalizing nanoparticles with targeting ligands or responsive moieties, researchers can customize their behavior and optimize their therapeutic performance for individual patients^[1]^. One of the most significant advantages of customized nanoparticle-based therapies is their ability to overcome the limitations of traditional drug delivery systems. Conventional medications often exhibit non-specific distribution, limited bioavailability, and systemic toxicity, leading to suboptimal therapeutic outcomes and adverse effects. In contrast, nanoparticles can be designed to selectively accumulate at disease sites, such as tumors or inflamed tissues, while sparing healthy cells, thereby maximizing drug efficacy and minimizing off-target effects^[2]^. In cardiology, customized nanoparticle-based therapies are promising for treating CVDs, including atherosclerosis, MI, and heart failure. For example, researchers have developed nanoparticle-based drug delivery systems capable of targeting atherosclerotic plaques and delivering anti-inflammatory or antithrombotic agents directly to the site of vascular inflammation or thrombosis^[3]^. By selectively modulating the activity of disease-promoting pathways while minimizing systemic exposure, these targeted therapies have the potential to reduce plaque burden, stabilize vulnerable plaques, and prevent cardiovascular events. Similarly, customized nanoparticle-based therapies in regenerative medicine offer exciting opportunities for enhancing tissue repair and regeneration following cardiac injury. Nanoparticles can carry growth factors, cytokines, or nucleic acids that promote angiogenesis, tissue remodeling, and stem cell recruitment in damaged heart tissue^[4]^. By delivering bioactive molecules directly to the site of injury and modulating the local microenvironment, nanoparticles can stimulate endogenous repair mechanisms and enhance the regenerative capacity of the heart, ultimately improving cardiac function and preventing adverse remodeling. Another application of customized nanoparticle-based therapies in cardiology is the development of personalized diagnostic and imaging agents for precision medicine. Nanoparticles can be functionalized with targeting ligands, fluorescent dyes, or contrast agents to enable non-invasive imaging and detect specific molecular markers associated with CVDs^[5]^. By combining imaging modalities such as MRI, CT, or PET with nanoparticle-based contrast agents, clinicians can visualize pathological processes at the molecular level, assess disease severity, and monitor treatment response in real time. Despite the potential of customized nanoparticle-based therapies, several challenges remain to be addressed before their widespread clinical implementation. One of the key challenges is the development of robust and reproducible manufacturing processes for nanoparticles, ensuring their scalability, uniformity, and quality control. To achieve desired therapeutic outcomes, nanoparticle synthesis, functionalization, and characterization require precise control over various parameters, including particle size, shape, surface charge, and drug loading efficiency^[6]^. Another challenge is the optimization of nanoparticle formulations for efficient delivery and controlled release of therapeutic agents. Nanoparticle pharmacokinetics, biodistribution, and stability can be influenced by various factors, such as surface modifications, drug loading methods, and physiological conditions, which must be carefully optimized to maximize therapeutic efficacy and minimize toxicity^[7]^. Moreover, the long-term safety profile of nanoparticle-based therapies, including potential immunogenicity, off-target effects, and accumulation in non-target organs, requires thorough evaluation through preclinical and clinical studies to ensure patient safety^[8]^. Furthermore, the regulatory approval process for nanoparticle-based therapies presents unique challenges due to the complex nature of these nanoscale drug delivery systems. Regulatory agencies require comprehensive data on nanoparticle formulations’ safety, efficacy, and quality, as well as evidence of their clinical benefit compared to standard treatments^[9]^. Therefore, interdisciplinary collaborations between researchers, clinicians, regulators, and industry stakeholders are essential to navigate the regulatory pathway, address regulatory concerns, and facilitate the translation of customized nanoparticle-based therapies from bench to bedside^[10]^. Nanosensors represent a revolutionary tool for personalized medicine, offering the ability to monitor physiological parameters, biomarkers, and drug levels with unprecedented sensitivity and specificity. These miniature devices, typically constructed from nanomaterials such as CNT, QDs, or nanowires, can detect minute changes in biological signals and transmit real-time data to external monitoring systems^[1]^. By implanting or integrating nanosensors into wearable devices, smartphones, or remote monitoring platforms, clinicians can continuously monitor key indicators of cardiovascular health, such as blood pressure, heart rate, oxygen saturation, and biomarker levels, allowing for timely intervention and personalized treatment adjustments^[2]^. One of the primary benefits of personalized dosing and monitoring is the ability to optimize medication regimens for individual patients based on their unique physiological characteristics, disease states, and response profiles. Traditional dosing strategies often rely on standardized treatment protocols or population-based guidelines, which may not account for interindividual variability in drug metabolism, pharmacokinetics, or drug-drug interactions. By contrast, personalized dosing algorithms leverage real-time physiological data from nanosensors to calculate individualized dosages tailored to each patient’s needs, maximizing therapeutic efficacy while minimizing the risk of adverse effects^[3]^. For example, in the management of hypertension, personalized dosing, and monitoring systems can dynamically adjust antihypertensive medications based on changes in blood pressure levels, heart rate variability, or renal function, allowing for precise titration of drug doses to achieve optimal blood pressure control while avoiding hypotensive episodes or medication-related adverse effects^[4]^. Similarly, in patients with heart failure, nanosensor-based monitoring platforms can track parameters such as fluid status, cardiac output, and biomarker levels, enabling early detection of decompensation and guiding adjustments to diuretic therapy, vasodilators, or inotropic agents to prevent exacerbations and hospitalizations^[5]^. Furthermore, personalized dosing and monitoring strategies can improve medication adherence and patient engagement in self-care, leading to better treatment outcomes and reduced healthcare costs. Nanosensor-enabled monitoring systems can empower individuals to manage their health and adhere to prescribed treatment regimens by providing real-time feedback on their physiological parameters and medication adherence. Smartphone apps, wearable devices, and telehealth platforms equipped with nanosensor technology can deliver patients personalized reminders, alerts, and educational resources, fostering greater compliance and accountability in medication management^[6]^. In addition to optimizing medication dosing, personalized monitoring can facilitate early detection of treatment non-response or adverse reactions, allowing for timely intervention and adjustment of therapeutic strategies. For example, in patients receiving anticoagulant therapy for atrial fibrillation or venous thromboembolism, nanosensor-based monitoring of coagulation parameters, such as prothrombin time or international normalized ratio (INR), can identify individuals at increased risk of thrombotic events or bleeding complications, prompting dose adjustments or alternative treatment options^[7]^. Similarly, nanosensor-enabled wearable devices can track exercise tolerance, electrocardiographic changes, and biomarker levels in patients undergoing cardiac rehabilitation or remote monitoring following cardiovascular procedures, enabling early detection of postoperative complications or disease progression^[8]^. Despite the potential benefits, personalized dosing and monitoring face several challenges that must be addressed to realize their full clinical impact. One challenge is developing robust, reliable, cost-effective nanosensor technology suitable for widespread clinical use. Nanosensors must exhibit high sensitivity, specificity, and stability in physiological environments while minimizing interference from endogenous molecules or environmental factors. Furthermore, nanosensor-enabled monitoring platforms must be user-friendly, interoperable, and compliant with data privacy regulations to ensure patient acceptance and regulatory approval^[9]^. Another challenge is integrating nanosensor data into clinical decision-making processes and electronic health records (EHRs) to facilitate real-time feedback and personalized treatment recommendations. Clinicians require intuitive, customizable dashboards or software interfaces that can aggregate, analyze, and visualize nanosensor data in a clinically meaningful manner, enabling informed decision-making and timely interventions. Moreover, healthcare systems must implement infrastructure and workflows to support remote monitoring, telemedicine consultations, and virtual care delivery models that leverage nanosensor-enabled technologies^[10]^. Furthermore, the validation and standardization of personalized dosing algorithms and treatment protocols based on nanosensor data require extensive clinical trials and evidence-based guidelines. Large-scale multicenter studies are needed to assess the safety, efficacy, and cost-effectiveness of personalized dosing and monitoring strategies in diverse patient populations and clinical settings. Moreover, developing personalized dosing algorithms necessitates interdisciplinary collaboration between clinicians, pharmacologists, bioinformaticians, and engineers to integrate complex data analytics, ML algorithms, and clinical expertise into predictive models. These algorithms should incorporate patient-specific factors such as age, gender, genetic polymorphisms, comorbidities, and concomitant medications to generate tailored dosing recommendations that optimize therapeutic outcomes while minimizing the risk of adverse events. Furthermore, ongoing monitoring and iterative refinement of dosing algorithms based on real-world data and patient feedback are essential to ensure their accuracy, reliability, and generalizability across diverse patient populations^[11]^. Another challenge is the ethical, legal, and regulatory considerations surrounding nanosensor-enabled monitoring systems in clinical practice. Patient privacy, data security, and informed consent are paramount concerns that require robust safeguards and transparent policies to protect sensitive health information and ensure patient autonomy. Moreover, regulatory agencies such as the U.S. Food and Drug Administration (FDA) and the European Medicines Agency (EMA) need to establish clear guidelines and approval pathways for nanosensor-enabled medical devices, including validation of their safety, efficacy, and performance characteristics through rigorous preclinical and clinical testing^[12]^. Despite these challenges, personalized dosing and monitoring hold immense potential for transforming the management of chronic cardiovascular conditions and improving patient outcomes. By harnessing the power of nanotechnology-enabled sensors, clinicians can deliver precise, individualized treatments tailored to each patient’s unique physiology, disease state, and treatment response. This paradigm shift from one-size-fits-all to personalized medicine promises to revolutionize healthcare delivery, paving the way for more effective, efficient, and patient-centered approaches to cardiovascular care. Clinical examples and case studies provide valuable insights into the practical applications and potential benefits of customized nanoparticle-based therapies in real-world medical settings. By examining success stories, patient outcomes, and ongoing challenges, clinicians and researchers can better understand these innovative treatment modalities’ clinical utility and limitations. In cardiology, customized nanoparticle-based therapies promise to improve the management of CVDs such as atherosclerosis, MI, and heart failure. One clinical example involves using targeted nanoparticles loaded with statins to treat atherosclerotic plaques^[1]^. By encapsulating statins within nanoparticles functionalized with targeting ligands specific to activated endothelial cells or macrophages within atherosclerotic lesions, researchers can achieve site-specific drug delivery and enhance the therapeutic efficacy of statins while minimizing systemic side effects^[2]^. Clinical studies have demonstrated that nanoparticle-mediated delivery of statins can lead to regression of atherosclerotic plaques, reduced inflammation, and improved endothelial function, thereby reducing the risk of cardiovascular events such as heart attack and stroke^[3]^. Another clinical example is nanoparticle-based drug delivery systems for targeted therapy in patients with heart failure. Researchers have developed nanoparticles capable of delivering therapeutic peptides, growth factors, or gene therapies directly to the damaged myocardium to promote tissue repair and regeneration^[4]^. By encapsulating these bioactive agents within biocompatible nanoparticles and administering them via intracoronary infusion or intramyocardial injection, clinicians can enhance the regenerative capacity of the heart and improve cardiac function in patients with heart failure^[5]^. Clinical trials evaluating nanoparticle-based therapies for heart failure have shown promising results, with improved left ventricular function, exercise tolerance, and quality of life observed in treated patients^[6]^. In oncology, customized nanoparticle-based therapies have shown considerable potential for improving the efficacy and safety of cancer treatments. One clinical example is nanoparticle-based drug delivery systems to overcome multidrug resistance in chemotherapy-resistant tumors^[7]^. By encapsulating chemotherapeutic agents within nanoparticles coated with targeting ligands or stimuli-responsive polymers, researchers can bypass efflux transporters and drug-resistant mechanisms, restoring sensitivity to chemotherapy and enhancing tumor suppression^[8]^. Clinical trials investigating nanoparticle-based therapies in patients with multidrug-resistant cancers have demonstrated encouraging outcomes, including tumor regression, prolonged progression-free survival, and reduced systemic toxicity compared to conventional chemotherapy regimens^[9]^. Another clinical example is the application of nanoparticle-based immunotherapy for cancer treatment. Researchers have developed nanoparticles capable of delivering immunomodulatory agents such as cytokines, checkpoint inhibitors, or nucleic acids to tumor-infiltrating immune cells, thereby enhancing antitumor immune responses and inhibiting tumor growth^[10]^. By harnessing the unique properties of nanoparticles to modulate the tumor microenvironment and overcome immune evasion mechanisms, clinicians can augment the efficacy of immunotherapy and improve patient responses to treatment^[11]^. Clinical trials evaluating nanoparticle-based immunotherapies have shown promising results, with durable responses and long-term survival observed in patients with advanced or refractory cancers^[12]^. In neurology, customized nanoparticle-based therapies offer novel approaches for treating neurological disorders such as Alzheimer’s disease, Parkinson’s disease, and glioblastoma. One clinical example involves nanoparticle-based drug delivery systems to enhance blood-brain barrier penetration and target neurodegenerative pathways in Alzheimer’s disease^[16]^. By encapsulating neuroprotective agents or disease-modifying drugs within nanoparticles engineered to cross the blood-brain barrier via receptor-mediated transcytosis or active transport mechanisms, researchers can deliver therapeutic payloads directly to affected brain regions and mitigate neurotoxicity, inflammation, and protein aggregation^[13]^. Clinical trials investigating nanoparticle-based therapies for Alzheimer’s disease have shown promising results, with improved cognitive function, biomarker profiles, and neuroimaging parameters observed in treated patients^[14]^. Another clinical example is the use of nanoparticle-based gene therapies for treating glioblastoma, a highly aggressive form of brain cancer. Researchers have developed nanoparticles capable of delivering therapeutic genes, small interfering RNAs, or CRISPR-Cas9 gene editing tools to glioblastoma cells, thereby modulating key signaling pathways involved in tumor progression, angiogenesis, and immune evasion^[15]^. By exploiting the unique properties of nanoparticles to protect and deliver nucleic acids to intracellular targets, clinicians can enhance the specificity and efficacy of gene therapies for glioblastoma while minimizing off-target effects and systemic toxicity^[17]^. Clinical trials evaluating nanoparticle-based gene therapies in patients with glioblastoma have shown encouraging results, with tumor regression, prolonged survival, and improved quality of life observed in treated individuals^[18]^. Despite the promising clinical examples and case studies discussed above, several challenges remain to be addressed to realize the full potential of customized nanoparticle-based therapies in clinical practice. One challenge is the translation of preclinical findings into clinically viable formulations and delivery systems that meet regulatory standards for safety, efficacy, and quality^[19]^. Developing scalable manufacturing processes, optimizing nanoparticle formulations, and conducting rigorous preclinical and clinical testing are essential steps in the drug development pipeline to ensure successful clinical translation of nanoparticle-based therapies^[20]^. Another challenge is the heterogeneity of patient populations and disease phenotypes, which may necessitate personalized treatment approaches tailored to individual patient characteristics^[21]^. Designing clinical trials that account for patient variability, biomarker stratification, and treatment response heterogeneity is critical for identifying subpopulations most likely to benefit from nanoparticle-based therapies^[22]^. Moreover, integrating biomarker-based companion diagnostics into clinical practice can facilitate patient selection, treatment monitoring, and response prediction, enabling personalized medicine approaches in nanoparticle-based therapy^[23]^. Furthermore, nanoparticle-based therapies’ long-term safety and biocompatibility, particularly in chronic or elderly patients, require careful evaluation through post-marketing surveillance and pharmacovigilance studies^[24]^. Monitoring for potential off-target effects, immune reactions, or nanoparticle accumulation in non-target tissues is essential for ensuring patient safety and minimizing the risk of adverse events associated with nanoparticle-based treatments^[25]^.

## Challenges and limitations in the application of nanotechnology in cardiology

One of the primary challenges in applying nanotechnology in cardiology is ensuring the biocompatibility and safety of nanomaterials used in diagnostic and therapeutic applications. While nanomaterials offer unique properties and advantages, such as high surface area-to-volume ratio and tunable physicochemical properties, their interactions with biological systems can be complex and unpredictable. Concerns about nanomaterials’ potential toxicity, immunogenicity, and long-term effects on cardiovascular health have been raised. For example, certain nanoparticles may trigger inflammatory responses or cause oxidative stress, leading to adverse cardiovascular effects^[1]^. Additionally, the biocompatibility of nanomaterials may vary depending on factors such as size, shape, surface chemistry, and route of administration. Addressing biocompatibility and safety concerns in nanocardiology requires the development of strategies for mitigating risks and ensuring the safe use of nanomaterials in clinical settings. This may involve comprehensive toxicity screening studies using in vitro and in vivo models to assess the biological effects of nanomaterials on cardiovascular cells and tissues^[2]^.

Furthermore, developing biocompatible coatings or surface modifications can help minimize adverse reactions and improve the compatibility of nanomaterials with biological systems^[80]^. Rigorous preclinical evaluation and risk assessment are essential in identifying and mitigating potential hazards associated with nanomaterials, ultimately ensuring patient safety in nanocardiology applications^[2]^. Another significant challenge in applying nanotechnology in cardiology relates to regulatory hurdles and ethical considerations. Regulatory agencies such as the U.S. Food and Drug Administration (FDA) are critical in evaluating the safety and efficacy of medical devices and therapies, including those incorporating nanomaterials. However, the regulatory framework for nanotechnology-based medical products is still evolving, and there needs to be standardized guidelines and criteria for evaluating nanomedical devices and formulations^[81]^. The complex nature of nanomaterials poses challenges for traditional regulatory approaches, necessitating innovative strategies for risk assessment and regulatory oversight. Navigating the regulatory pathway for nanotechnology-enabled cardiac interventions requires close collaboration between researchers, clinicians, industry stakeholders, and regulatory agencies. It involves extensive preclinical testing, clinical trials, and regulatory submissions to demonstrate nanomedical products’ safety, efficacy, and quality^[82]^.

Moreover, ethical considerations surrounding the equitable distribution and affordability of nanomedicine in cardiology must be carefully addressed. Ensuring patient access to cutting-edge nanocardiology therapies while safeguarding against potential risks and exploitation is essential for promoting ethical and equitable healthcare delivery^[83]^. In addition to biocompatibility and regulatory issues, scalability and manufacturing challenges pose significant obstacles to the widespread adoption of nanotechnology in cardiology. While nanomaterial synthesis and fabrication techniques have advanced significantly in recent years, scaling up production to meet clinical demand remains a major challenge. Nanoparticle manufacturing processes often require specialized equipment, expertise, and resources, leading to high production costs and limited scalability^[84]^.

Moreover, the variability and complexity of nanomaterial properties can pose challenges to quality control and reproducibility in large-scale manufacturing. Addressing scalability and manufacturing challenges in nanocardiology requires concerted efforts from academia, industry, and government agencies to optimize production processes, reduce costs, and improve efficiency. This may involve the development of novel fabrication methods, automation technologies, and process optimization strategies to streamline nanoparticle manufacturing and scale up production^[85]^. Collaboration between researchers and industry partners is essential for translating laboratory-scale synthesis techniques into robust, scalable manufacturing processes suitable for commercialization. Furthermore, regulatory agencies play a crucial role in establishing guidelines and standards for quality control, safety, and consistency in nanoparticle manufacturing, ensuring the reliability and reproducibility of nanocardiology products^[86]^.

## Side effects and limitations of nanotechnology in cardiology

Nanoparticles, the central elements in nanotechnology, can interact with biological systems in complex and sometimes unpredictable ways. Their small size and large surface area allow them to penetrate biological barriers and target specific tissues, which is one of the key advantages of nanotechnology^[1-3]^. However, these properties can lead to unintended and potentially harmful biological effects. One of the nanoparticles’ most significant side effects is their potential to induce immune responses, ranging from mild inflammation to severe hypersensitivity reactions. Studies have demonstrated that the physicochemical properties of nanoparticles, such as size, shape, surface charge, and chemical composition, can influence the type and severity of immune responses. For example, gold and silver nanoparticles are known to activate immune cells like macrophages and dendritic cells, resulting in the release of pro-inflammatory cytokines^[4-6]^. These immune reactions can exacerbate pre-existing cardiovascular conditions, such as atherosclerosis and MI, leading to further complications in patients undergoing treatment. Furthermore, chronic inflammation resulting from nanoparticle exposure may contribute to the development of CVDs, further complicating patient outcomes.

Another major concern with using nanotechnology in cardiology is the issue of off-target effects. While nanoparticles are designed to target specific tissues or cells, their biodistribution and accumulation within the body are often unpredictable. As a result, nanoparticles can accumulate in non-target organs, such as the liver, spleen, or kidneys, leading to unintended toxicities^[7-9]^. For instance, polyethylene glycol (PEG)-coated nanoparticles, commonly used to improve the biocompatibility and circulation time of nanoparticles, have been shown to accumulate in the spleen, leading to splenomegaly in animal models. These off-target effects raise significant concerns about the long-term safety of nanotechnology-based interventions in cardiology, as the inadvertent accumulation of nanoparticles in non-target organs could result in organ dysfunction and tissue damage. The unpredictable nature of nanoparticle biodistribution necessitates further research into improving their targeting efficiency and minimizing off-target effects^[10-12]^.

In addition to immune responses and off-target effects, toxicity remains one of the most significant limitations of nanotechnology in cardiology. Different types of nanoparticles, including metallic, polymeric, and lipid-based nanoparticles, have been associated with a range of cytotoxic and genotoxic effects. The toxicity of nanoparticles is primarily linked to their ability to generate reactive oxygen species, which can cause oxidative stress and damage to cellular components such as lipids, proteins, and DNA^[13-16]^. For instance, iron oxide nanoparticles, commonly used in imaging applications, have been shown to induce mitochondrial dysfunction and apoptosis in cardiomyocytes. Similarly, carbon-based nanoparticles, such as graphene and CNT, have been reported to cause DNA strand breaks and chromosomal aberrations in vitro^[15]^. These genotoxic effects are particularly concerning when considering the long-term nature of many cardiovascular treatments, as prolonged exposure to toxic nanoparticles may increase the risk of chronic conditions like fibrosis, carcinogenesis, and cardiomyopathy. As such, further studies are needed to assess the full extent of nanoparticle-induced toxicity in the context of CVDs and to develop safer alternatives for clinical use^[17]^.

Despite the promising potential of nanotechnology in cardiology, its widespread clinical adoption is hindered by several production and scalability challenges. The synthesis of nanoparticles with consistent size, shape, and functionalization remains a complex and resource-intensive process. Even small variations in these parameters can significantly alter the pharmacokinetics, biodistribution, and therapeutic efficacy of nanoparticles^[18-20]^. Achieving consistent quality control in nanoparticle production is particularly challenging when scaling from laboratory conditions to industrial-scale manufacturing. The process of nanoparticle fabrication often requires expensive raw materials, sophisticated equipment, and specialized expertise, which can drive up the cost of production. These challenges make translating laboratory-scale nanotechnology innovations into clinically viable treatments difficult. As a result, many promising nanoparticle-based therapies remain limited to research settings, and the lack of scalable production techniques prevents their widespread adoption in clinical practice^[21-23]^.

The lack of standardization in the characterization and evaluation of nanoparticles is another significant limitation of nanotechnology in cardiology. There is currently no universally accepted set of guidelines for the characterization of nanoparticles, which leads to inconsistencies in the data reported in the literature. Variations in the methods used to assess nanoparticle properties, such as size, surface charge, and stability, make it difficult to compare studies and establish reliable safety profiles^[24-26]^. Inconsistent characterization can also lead to discrepancies in the reported therapeutic efficacy of nanoparticles, complicating the decision-making process for clinicians. Moreover, the lack of a standardized approach to nanoparticle characterization raises concerns about the reproducibility of results, particularly when nanoparticle-based therapies are transferred from preclinical models to clinical applications. The absence of consistent characterization methods further complicates the regulatory approval process, as regulators often rely on well-defined standards to assess the safety and efficacy of medical products^[27]^.

The regulatory landscape for nanotechnology-based medical products is still evolving, and regulatory uncertainty poses a significant barrier to the widespread clinical use of nanoparticles in cardiology. Traditional regulatory frameworks for medical devices and therapies are often ill-equipped to address the unique challenges posed by nanomaterials. Unlike conventional drugs or devices, nanoparticles exhibit distinct properties, such as their small size and ability to cross biological barriers, which require specialized evaluation methods. However, there is currently no comprehensive regulatory framework in place to assess the safety, efficacy, and quality of nanotechnology-based cardiovascular treatments^[28-30]^. This regulatory gap has resulted in a patchwork of inconsistent guidelines across different regions, further complicating the commercialization of nanomedicines. To facilitate the global adoption of nanotechnology in cardiology, there is a need to develop clear, standardized guidelines for the approval of nanotechnology-based medical products and international collaboration to harmonize regulatory processes^[31]^.

In addition to regulatory challenges, ethical concerns play a crucial role in deploying nanotechnology in cardiology. The use of nanoparticles in medical treatments raises several ethical questions, particularly regarding patient autonomy, informed consent, and the equitable distribution of healthcare resources. Patients may not fully understand the potential risks and benefits of nanoparticle-based therapies, especially since the long-term effects of these treatments are still not well understood^[32-34]^. Furthermore, the high cost of nanotechnology-based interventions raises concerns about access to care, particularly in low- and middle-income countries with limited resources for advanced medical technologies. Ethical considerations surrounding the affordability and accessibility of nanotechnology-based treatments must be carefully addressed to ensure that these innovations benefit all patients, regardless of socioeconomic status^[35]^.

The environmental impact of nanotechnology is another important consideration. Nanoparticles released into the environment during their production, use, or disposal can accumulate in soil, water, and air, posing risks to ecosystems and human health. For instance, silver nanoparticles, which are commonly used for their antimicrobial properties, have been shown to disrupt aquatic ecosystems by inhibiting the growth of beneficial microorganisms^[36-38]^. These environmental concerns highlight the need for sustainable manufacturing practices and safe disposal methods for nanomaterials. Developing environmentally friendly approaches to nanoparticle synthesis and disposal will be essential for minimizing the ecological footprint of nanotechnology and ensuring its long-term sustainability^[39]^.

The in vivo behavior of nanoparticles is highly complex and can vary depending on factors such as particle size, surface charge, and protein corona formation. Nanoparticles are often coated with proteins upon entering the body, forming a “protein corona” that can alter their biological activity. This protein corona can mask the functional groups of nanoparticles, hindering their ability to target specific cells or tissues^[40-42]^. Additionally, the mononuclear phagocyte system (MPS) often clears nanoparticles from the bloodstream, which can significantly reduce their circulation time and therapeutic efficacy. Overcoming these challenges requires advanced engineering strategies to optimize the design and functionality of nanoparticles, ensuring that they reach their intended targets and remain effective over time^[43-45]^.

Lastly, the long-term effects of nanoparticle exposure remain largely unknown. Most preclinical and clinical studies focus on the short-term outcomes of nanoparticle-based treatments, leaving gaps in our understanding of the chronic effects of these therapies. It is still unclear whether nanoparticles accumulate in tissues over time and whether they could cause delayed toxicities, such as fibrosis, carcinogenesis, or neurological damage. To address these knowledge gaps, further research is needed, including long-term studies and post-market surveillance, to monitor the safety of nanotechnology-based treatments in cardiology and other medical fields^[46-48]^.

## Final remarks

The application of nanotechnology in cardiology represents a promising frontier for advancing diagnostic techniques, therapeutic interventions, and patient care. Through the development of novel nanomaterials, drug delivery systems, imaging agents, and tissue engineering approaches, researchers and clinicians have the potential to revolutionize the diagnosis, treatment, and prevention of CVDs. However, despite the significant progress made in nanocardiology, several challenges and barriers still need to be addressed. Biocompatibility and safety concerns, regulatory hurdles, scalability issues, and ethical considerations must be carefully navigated to ensure nanotechnology’s safe and effective implementation in clinical practice. As we look to the future, stakeholders across the healthcare landscape must remain committed to advancing nanocardiology and harnessing its transformative potential. By working together and embracing the opportunities offered by nanotechnology, we can usher in a new era of personalized, precision medicine to prevent and treat CVDs.

## Data Availability

This published article and its supplementary information files include all data generated or analyzed during this study.
